# Multifunctional IgG/IgM antibodies and cellular cytotoxicity are elicited by the full-length MSP1 SumayaVac-1 malaria vaccine

**DOI:** 10.1038/s41541-023-00701-2

**Published:** 2023-08-09

**Authors:** Micha Rosenkranz, Kristin Fürle, Julia Hibbert, Anne Ulmer, Arin Ali, Thomas Giese, Antje Blank, Walter E. Haefeli, Ernst Böhnlein, Michael Lanzer, Richard Thomson-Luque

**Affiliations:** 1 Center for Infectious Diseases-Parasitology, Heidelberg University Hospital, Heidelberg, Germany; 2 Institute for Immunology, Heidelberg University Hospital and German Center for Infection Research (DZIF), Heidelberg, Germany; 3 Clinical Pharmacology and Pharmacoepidemiology, Heidelberg University Hospital, Heidelberg, Germany; 4 Sumaya-Biotech GmbH & Co. KG, Heidelberg, Germany

**Keywords:** Malaria, Protein vaccines

## Abstract

Radical control of malaria likely requires a vaccine that targets both the asymptomatic liver stages and the disease-causing blood stages of the human malaria parasite *Plasmodium falciparum*. While substantial progress has been made towards liver stage vaccines, the development of a blood stage vaccine is lagging behind. We have recently conducted a first-in-human clinical trial to evaluate the safety and immunogenicity of the recombinant, full-length merozoite surface protein 1 (MSP1_FL_) formulated with GLA-SE as adjuvant. Here, we show that the vaccine, termed *SumayaVac-1*, elicited both a humoral and cellular immune response as well as a recall T cell memory. The induced IgG and IgM antibodies were able to stimulate various Fc-mediated effector mechanisms associated with protection against malaria, including phagocytosis, release of reactive oxygen species, production of IFN-γ as well as complement activation and fixation. The multifunctional activity of the humoral immune response remained for at least 6 months after vaccination and was comparable to that of naturally acquired anti-MSP1 antibodies from semi-immune adults from Kenya. We further present evidence of *SumayaVac-1* eliciting a recallable cellular cytotoxicity by IFN-γ producing CD8+ T cells. Our study revitalizes MSP1_FL_ as a relevant blood stage vaccine candidate and warrants further evaluation of *SumayaVac-1* in a phase II efficacy trial.

## Introduction

Recent years have witnessed an increase in malaria incidence and mortality to an estimated 247 million clinical cases and 619,000 deaths as of 2021^1^. Apparently, the currently deployed intervention strategies^2^, i.e., vector control, chemotherapy and chemoprophylaxis, are insufficient to reduce sustainably the malaria burden, which, in turn, accentuates the need for an effective malaria vaccine, in particular one that protects against the most virulent form caused by the protozoan parasite *Plasmodium falciparum*. However, the development of an efficacious malaria vaccine has turned out to be complicated, partly because of the complex life cycle of the parasite and a long history of co-evolutionary adaptation with the human host. Nevertheless, progress has been made, particularly towards vaccines that specifically target the liver stages. This includes RTS,S (MosquirixTM, GSK Bio), which was recommended by the WHO in 2021, but offers only a moderate efficacy in infants aged 6–12 weeks, the age group most vulnerable to malaria^3^. More efficacious seems to be the vaccine candidate R21 that reached the WHO-specified malaria vaccine efficacy goal of 75% protection against severe malaria in African children^4^ in a phase 2 clinical trial^5,6^. Protection against *P. falciparum* malaria was also demonstrated in clinical trials using attenuated sporozoites (the stage transmitted by Anopheles mosquitoes during blood feeding) as the drug product (Sanaria´s PfSPZ)^7,8^.

Liver stage vaccines have the advantage of targeting the parasite from the moment it is transmitted by the bite of an Anopheline mosquito until the parasite has completed its development in hepatocytes^9^. However, liver stage vaccines offer no or very little protection against the subsequent asexual blood stages that cause most of the pathology associated with *P. falciparum* malaria, including anaemia, hypoglycaemia, vaso-occlusive events and the syndromes associated with maternal and cerebral malaria^10^. As a consequence, any parasite leaving the liver will likely escape the pre-erythrocytic vaccine cover and may cause symptomatic, life-threatening disease if left untreated. It is therefore desirable to combine a liver-and a blood-stage vaccine.

Efforts to develop a blood stage vaccine have been sobering, in spite of encouraging immune-epidemiological studies showing that residents from malaria endemic areas are able to attain, with time and after repeated exposure to *P. falciparum* infections, a strain-transcending antigenic memory that protects against clinical disease^11^. A key component of this premunition are antibodies as demonstrated by passive immunization studies using IgG antibodies^12^. Antibodies can induce multiple effector mechanisms to control asexual blood stage development of the parasite and, hence, disease manifestation. For example, antibodies can directly inhibit invasion of erythrocytes by merozoites in a Fab-dependent manner, and the subsequent intraerythrocytic development^13–16^. The blood stage PfRH5-based vaccine candidate^17^ is thought to utilize this mechanism to reduce parasitemia. PfRH5^17^ is an advanced vaccine, which is going through an encouraging trajectory of clinical development that has led to higher levels of neutralization in the growth inhibition assay (GIA)^18–20^ and reduced blood-stage malaria growth, but no protection yet in a controlled human malaria infection (CHMI)^21^. In addition, antibodies can activate complement^22,23^ and induce Fc-receptor-dependent killing mechanisms, including monocyte^24,25^ and neutrophil-mediated^26–28^ phagocytosis, degranulation and interferon gamma (IFN-γ) production of natural killer (NK) cells^29^ as well as release of reactive oxygen species (ROS) by neutrophils^26^. These Fc-mediated effector functions correlate stronger with protection^30–32^ than the GIA, frequently used to determine the neutralization potential of antibodies^32–36^.

Since merozoites are directly accessible by the immune system and given that antibodies against merozoites can control disease progression^37^, considerable effort has been devoted to the identification of protective blood stage antigens. The first merozoite antigen discovered was the merozoite surface protein 1 (MSP1). The 196 kDa MSP1 is the most abundant protein on the merozoite surface^38^. During merozoite maturation, it is processed by subtilisin-like proteases into four subunits, termed: p83, p30, p38 and p42^38,39^. The subunits remain attached to one another and are anchored to the plasma membrane via a glycosylphosphatidylinositol (GPI) moiety. Cleavage of MSP1 activates a spectrin binding function that destabilizes the membrane skeleton of the infected erythrocytes, thereby contributing to cell rupture and merozoite egress^40^. The MSP1 complex recruits variable factors to form a supramolecular structure^41–43^ that interacts with molecules on the surface of the erythrocyte during invasion^44,45^. As the merozoite invades the erythrocyte, MSP1 is shed via cleavage within the p42 subunit^46^, leaving only the GPI anchored p19 subunit^47^ attached to the merozoite.

Some, but not all studies found a correlation between antibodies against MSP1 and protection from malaria^48–53^. Recently, a CHMI study in semi-immune adults showed that MSP1 can induce multiple effector functions that correlated with protection against malaria^32,54^. Even more encouraging, MSP1-based vaccines were effective in animal models, such as mice^55,56^ and non-human primates^57–60^ and were able to elicit sterile immunity or could control or delay the infection from progressing. In contrast, MSP1-derived vaccines performed poorly in humans^61,62^. However, unlike the studies conducted in animal models, none of the human trials vaccinated full-length MSP1 (MSP1_FL_). Instead, they used the p42 or p19 subunits^63,64^ or composites consisting of various small domains of MSP1^65,66^. Thus, the entire MSP1 was never tested in human clinical trials in spite of encouraging results in non-human primates and the fact that the remaining MSP1 subunits harbor multiple B- and T cell epitopes^54,67–73^ that can potentially contribute to anti-parasitic effector mechanisms^54^.

We have recently conducted a first-in-human clinical trial of MSP1_FL_ formulated with the TLR4 agonist glucopyranosyl lipid A in a stable oil-in-water nanoemulsion (GLA-SE) as adjuvant *(SumayaVac-1*)^74^. A total of 32 volunteers were enrolled in the study, of which 24 volunteers received 3 or 4 injections of the MSP1_FL_ vaccine. *SumayaVac-1* was safe and immunogenic, with all 24 vaccinees seroconverting independent of the dose administered. Both IgG and IgM antibody titers peaked at levels exceeding those observed for sera from semi-immune individuals from Kenia at day 85, four weeks after the third vaccination. High antibody titers persisted for more than half a year and could be boosted by a fourth vaccination.

One of the major stumble blocks with blood stage vaccines is cross-strain variability and lack of heterologous protection. MSP1 is a dimorphic protein characterized by two prototypic sequences: the MSP1-D from the *P. falciparum* MAD20 strain and MSP1-F from the WELLCOME strain^75,76^. These two forms are found in African populations, with distinct distributions observed between East and West Africa. Importantly, human antibodies against MSP1-D generated by *SumayaVac-1* cross-reacted with MSP1-F and production of ROS in the ADRB assay was independent of the parasite strains 3D7 and FCB1 used^74^.

A fine-scale epitope mapping supported the previous assumption of the antigenic repertoire extending across the entire MSP1 molecule. Moreover, the induced functional antibodies could fix complement and opsonize merozoites to stimulate neutrophils and to release ROS. The vaccine further elicited a memory T cell responses as shown in the ELISpot assay (enzyme‐linked immunosorbent spot‐forming cell assay) of peripheral blood mononuclear cells (PBMCs) re-stimulated with MSP1_FL_.

Here, we have reexamined the humoral and cellular immune responses elicited by MSP1_FL_ in vaccinees, with a focus on functional immune effector mechanisms and the role both IgG and IgM play in activating different branches of the immune system. We show that both IgG and IgM from MSP1_FL_ vaccinees are able to activate the classical complement pathway, stimulate opsonic phagocytosis and trigger IFN-γ production and degranulation of NK cells. Moreover, we found that antibodies against the conserved N-terminal p83 subunit, never included in previous MSP1 trials, exhibited a high degree of functionality. Finally, we demonstrate the capacity of *SumayaVac-1* to elicit a recall memory T cell response and induce cellular cytotoxicity through CD8^+^ T cells.

## Results

### IgG from MSP1_FL_ vaccinees stimulate several Fc-mediated effector functions

**Fig. 1:**
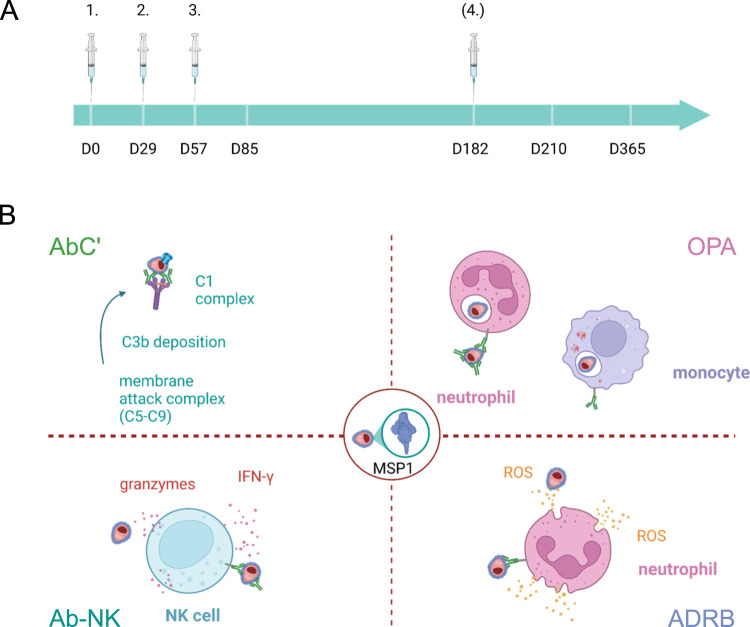
Illustration of antibody mediated effector functions against MSP1. **A** Immunization scheme of SumayaVac-1 (full length MSP1 plus GLA-SE as adjuvant) on days 0, 29, 57, and optionally after unblinding of the cohort on day 182. Blood samples were taken for serological analysis on the days indicated. The safety follow-up was scheduled 6 months after the last vaccination. **B** Illustration of antibody mediated effector functions against MSP1. Abbreviations: antibody dependent complement activity assays (AbC´), opsonic phagocytosis assay (OPA), antibody-dependent natural killer cell activity assay (Ab-NK), interferon (IFN), reactive oxygen species (ROS), antibody-dependent respiratory burst activity assay (ADRB) and merozoite surface protein 1 (MSP1).

Previous findings have revealed that the total number of functional Fc-mediated immune responses can predict the disease outcome^32,54^. We therefore tested the functionality of IgG from MSP1_FL_ vaccinees in a broad panel of Fc-mediated effector assays, including the opsonic phagocytosis assay (OPA)^28,54,77^, the antibody-dependent respiratory burst assay (ADRB)^26^, the antibody-dependent natural killer cell assay (Ab-NK)^29,78^, and the complement activity assay (AbC’)^22^ (Fig. 1).

**Fig. 2:**
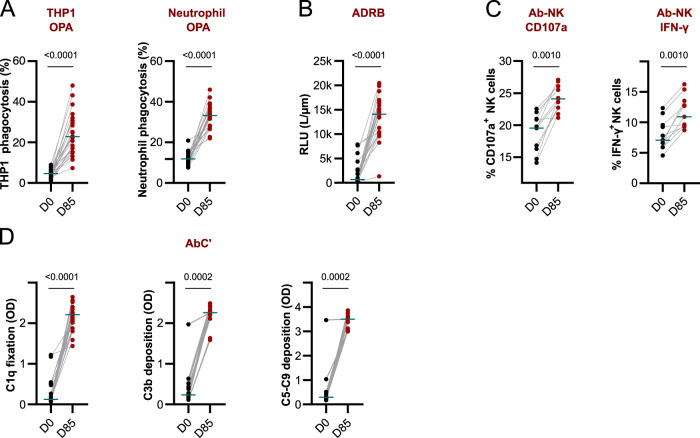
Fc-mediated effector mechanisms of vaccine-induced IgG. **A**–**D** Levels of Fc-mediated effector functions against MSP1_FL_ of purified IgG samples (*n* = 24, *n* = 11 for Ab-NK) were compared between before (D0) and after full immunization (D85). **A** opsonic phagocytosis activity (OPA) of MSP1_FL_-coupled microsphere beads by THP1 cells or neutrophils. **B** Antibody- dependent respiratory burst (ADRB) of neutrophils. **C** Antibody-dependent natural killer cell (Ab-NK) activity measured by degranulation (CD107a) and IFN-γ production. **D** Levels of antibody-dependent complement fixation (AbC’) measured by C1q fixation, C3b deposition and C5-C9 deposition (membrane attack complex, MAC). Each data point represents functional activity for one sample in duplicates. Statistical differences between timepoints were calculated using Wilcoxon test.

Purified IgG from MSP1_FL_ vaccinees collected at day 85, four weeks after the third immunization (Fig. 1), induced phagocytosis of MSP1_FL_-coated fluorescent microspheres by both the monocyte cell line THP1 (*p* < 0.0001) and neutrophils (*p* < 0.0001) in the opsonic phagocytosis assay (Fig. 2A, Supplementary Data 1). MSP1_FL_-coated microspheres yielded results comparable to those of merozoites (Supplementary Fig. 1). Purified IgG collected from vaccinees at time point 0 were analyzed in parallel and served as negative background-indicating controls. Purified IgG from MSP1_FL_ vaccinees could also activate neutrophils to release a respiratory burst in the ADRB assay (*p* < 0.0001) (Fig. 2B, Supplementary Data 1), consistent with previous reports^74^. Likewise, purified IgG from MSP1_FL_ vaccines stimulated NK cells in the Ab-NK assay as evidenced by an increased percentage of CD107a^+^ cells, as a marker of degranulation, and enhanced levels of IFN-γ production, as compared with pre-immunization controls (*p* = 0.001 and *p* = 0.001 respectively) (Fig. 2C^,^ Supplementary Data 1). Furthermore, purified IgG from MSP1_FL_ vaccines activated the classical complement pathway as indicated by C1q fixation and C3b deposition (*p* < 0.0001 and *p* = 0.0002, respectively) and the formation of the membrane attack complex (C5-C9 deposition) (*p* = 0.0002) (Fig. 2D, Supplementary Data 1). The role of complement in this assay was previously verified, using heat-inactivated serum (Supplementary Fig. 1). In summary, purified IgG from vaccinees collected at day 85 could stimulate at least four different branches of Fc-mediated immune effector mechanisms. The functional activities were independent of the MSP1_FL_ dose administered (Supplementary Fig. 2).

### IgG titers correlate with Fc-mediated effector functions

**Fig. 3:**
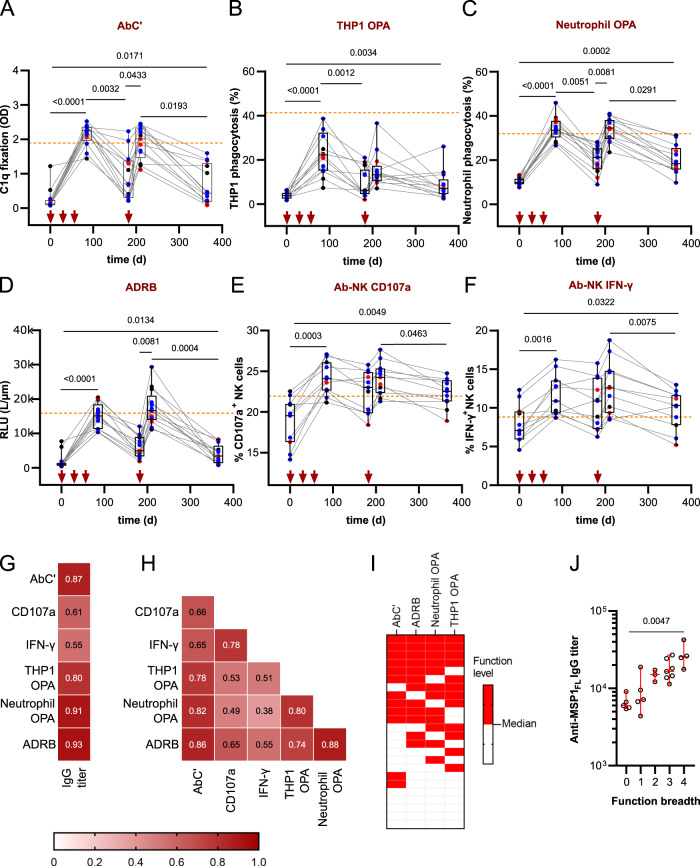
Kinetics and breadth of functional activity. **A**–**F** Levels of MSP1_FL_-specific Fc- mediated effector mechanisms of IgG (*n* = 13, *n* = 11 for Ab-NK) over time. **A** AbC‘; antibody- dependent complement fixation activity measured by C1q fixation. **B** OPA; opsonic phagocytosis activity of MSP1_FL_-coupled microsphere beads by THP1 cells or (**C**) neutrophils. **D** ADRB; antibody-dependent respiratory burst by neutrophils. **E** Ab-NK:CD107a; antibody- dependent NK cell degranulation (**F**) Ab-NK: IFN-γ; antibody-dependent NK cell IFN-γ production. **G** Heatmaps showing spearman correlations between IgG titers and effector functions and (**H**) effector functions with each other. Significant (*p* < 0.0001) spearman rank correlation coefficients for samples (*n* = 13) are highlighted in double digits. The colour intensity represents the strength of correlation. **I** A heatmap showing the activity levels of four Fc- mediated effector functions (AbC’, ADRB, THP1 OPA, neutrophils OPA) in vaccinees (*n* = 24) at day 85, four weeks after the last immunization. Responses above a function-specific median are shown in red. Each column is a Fc-mediated function while each row is a single volunteer. **J** Anti-MSP1_FL_ IgG titers post-immunization (D85) were compared between volunteers with different function breadth scores. Each data point represents functional activity for one sample in duplicates. Box plots show the median with min/max values. Colors of the dots represent different dose cohorts: red; 25 µg (*n* = 3), blue; 50 µg (*n* = 7), black; 150 µg (*n* = 3). Red arrows indicate the days of immunization. The dashed line indicates reference effector functions of purified IgG from pooled sera from semi-immune individuals from Kisumu, Kenya (NIBSC code 10/198). Statistical differences between timepoints were calculated using Friedman test and Wilcoxon ranked test; and Kruskal-Wallis between titers and breadth scores. For correlations Spearman rank test was used.

We next assessed the induction and decay kinetics of IgG-mediated effector functions in vaccines over a one-year follow-up period. We observed that the readouts of all functional assays peaked at day 85, sometimes exceeding the activity levels of semi-immune Kenyan adults (WHO control) (Fig. 3A–F, Supplementary Data 2), and then declined over a 3-month period, but peaked again at day 210, four weeks after a booster immunization on day 182. The increase in functional activity after the booster was significant for C1q fixation (*p* = 0.0433) (Fig. 3A), neutrophil OPA (*p* = 0.081) (Fig. 3C) and ADRB (*p* = 0.081) (Fig. 3D and Supplementary Data 2). The activity levels gradually declined following the last immunization, but remained significantly above baseline values for at least one year (AbC’ *p* = 0.0171. THP1 OPA *p* = 0.0034, neutrophil OPA *p* = 0.0002, ADRB *p* = 0.0134, Ab-NK: IFN-γ *p* = 0.0049 and Ab-NK:CD107a *p* = 0.0322) (Fig. 3A–F and Supplementary Data 2). Again, the effector functions were independent of the MSP1_FL_ dose vaccinated.

We noted that the dynamics in activity levels followed the up and downs of the previously published IgG titers^74^, suggesting a direct correlation. This was indeed the case, according to a Spearman rank order correlation for AbC’ (*r* = 0.87, 95% CI 0.7830 to 0.9236, *p* < 0.0001), THP1 OPA (*r* = 0.80, 95% CI 0.6778 to 0.8820, *p* < 0.0001), neutrophil OPA (*r* = 0.91, 95% CI 0.8443 to 0.9463, *p* < 0.0001), ADRB (*r* = 0.93, 95% CI 0.8834 to 0.9604, *p* < 0.0001), Ab-NK:CD107a (*r* = 0.61, 95% CI 0.3297 to 0.7173, *p* < 0.0001), and Ab-NK: IFN-γ (*r* = 0.55, 95% CI 0.3982 to 0.7536, *p* < 0.0001) (Fig. 3G, Supplementary Data 2).

We further investigated the correlation among the various assays and readout parameters and observed the highest level of cross-correlation between C1q fixation (AbC’), THP1 OPA, neutrophil OPA and ADRB (*r* = 0.74–0.88, *p* < 0.0001) and moderate correlation with the two Ab- NK activities (*r* = 0.38–0.65, *p* < 0.0001) (Fig. 3H, Supplementary Data 2). As expected, the two readouts of the Ab-NK assay correlated highly with each other (*r* = 0.78, 95% CI 0.6508 to 0.8708, *p* < 0.0001). We next categorized vaccinees into either “high responders” or “low responders” based on the calculated median of the various assays performed. As highlighted in Fig. 3I, IgG from vaccinees showing functionality in one assay were likely to activate other Fc-mediated immune responses. Furthermore, vaccinees with multifunctional Fc-mediated immune responses were also those having the highest IgG titers after the third vaccination (*p* = 0.0047) (Fig. 3J, Supplementary Data 2).

### N-terminal MSP1 p83 subunits leads to highly multifunctional IgGs

**Fig. 4:**
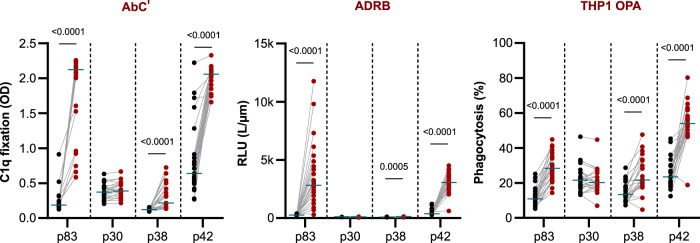
Functional activity of IgG against MSP1 subunits. Levels of functional activity AbC’, ADRB and THP1 OPA) of IgG samples (*n* = 23) (before (black dots) and after full immunization (red dots). Statistical differences between timepoints were calculated using Wilcoxon test.

Previously, we mapped B cell epitopes to all four naturally-generated subunits of MSP1 - p83, p30, p38 and p42^74^. On the basis of this finding, we explored the contribution of each of the subunits (all included in our MSP1_FL_ vaccine), to the various Fc-mediated immune responses. To this end, fluorescent microspheres as well as plates were coated with each of the four subunits before the reactivity in the various assays was tested in the presence of purified IgG from the MSP1_FL_ vaccinees. As shown in Fig. 4, we found significant and strong effector functions to the p83 and p42 subunits in the AbC’ (*p* < 0.0001), ADRB (*p* < 0.0001) and OPA (*p* < 0.0001) assay. Significant effector functions to p38 were observed in the AbC’ (*p* < 0.0001) and OPA assay (*p* < 0.0001) and to a lower extent in the ADRB assay (*p* = 0.0005). In comparison, reactivity to p30 was negligible (Fig. 4, Supplementary Fig. 4 and Supplementary Data 3).

### Antibody-mediated effector functions show cross-strain variability

Previously, we have shown that IgG from vaccinees cross-reacted with MSP1-F and they further activated neutrophils to release a respiratory burst in the ADRB assay when opsonizing *P. falciparum* merozoites from the 3D7 homologous strain as well as from the FCB1 heterologous strain^74^. To further investigate the strain-transcending nature of Fc- mediated effector functions triggered after immunization with *SumayaVac-1*, we compared the ADRB activities for 3 dosages previously obtained for 3D7 and FCB1 at group level and found the data to moderately correlate (*r* = 0.70, *p* = 0.0067) (Supplementary Fig. 3 and Supplementary Data 1). Furthermore, the ADRB activity using the FCB1 strain also correlated significantly with IgG titers from the volunteers (*r* = 0.57, *p* = 0.0366) (Supplementary Fig. 3 and Supplementary Data 1), as did ADRB activity and IgG titers when using the homologous 3D7 strain^74^. Moreover, we have now conducted a side-by-side comparison of two other representative Fc-mediated immune responses using the prototypic MSP1-D and MSP1-F in parallel. We found that antibodies from vaccinees post-immunization at day 85 were also functional in the OPA and C1q assay when the assays were performed with MSP1-F. Using fluorescent microspheres coupled with MSP1-F we found a significant increase (*p* < 0.0001) in the percent of phagocytosis by THP1. The OPA activity using MSP1-D and MSP1-F was also significantly correlated (*r* = 0.6387, *p* = 0.0091). Similar results were observed for C1q fixation with a trend in the correlation when MSP1-D and MSP1-F assays were compared. Both OPA activity and C1q fixation using MSP1-F were significantly correlated with IgG titers (*r* = 0.7441, *p* = 0.0014 for OPA and *r* = 0.5147, *p* = 0.0436 for C1q) as for ADRB using FCB1 (Supplementary Fig. 3 and Supplementary Data 1).

### IgM antibodies recognize multiple epitopes throughout the entire MSP1_FL_

**Fig. 5:**
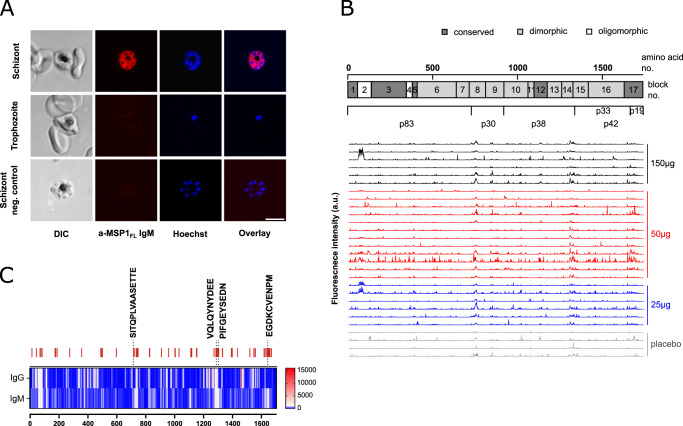
IgM epitope mapping. **A** Representative indirect immuno-fluorescence assay (IFA showing reactivity of IgM from an individual immunized 3× with *SumayaVac-1* with developing merozoites during schizogony and free mature merozoites. Trophozoites as well as non-IgM opsonized schizonts served as a negative control. Scale bar, 5 μm. **B** Mapping of linear B cell epitopes across MSP1 for IgM. Sera from vaccinees (1:1000) was applied to a custom-made MSP1 peptide microarray chip. The fluorescence intensity landscapes across MSP1 are shown for each sample (*n* = 24). Controls comprise the placebos (*n* = 4). For orientation, the structural organization of MSP1 is shown over the fluorescence profiles. Pre-staining was done with the respective secondary antibodies goat anti-human IgG (Fc) DyLight680, goat anti-human IgM (μ chain) DyLight800 (0.2 μg/ml). Full details of epitopes and fluorescence intensities are provided in the Data file S4. **C** Location over the entire MSP1 protein of the top 5% binder epitopes showing the stronger increases in the averaged median intensities between placebo (*n* = 4) and vaccinees (*n* = 24) for IgM. Relevant epitopes have been highlighted.

We have previously shown that vaccination with MSP1_FL_ mounted high, long-lasting IgM titers that reached levels comparable to those observed in semi-immune individuals from Burkina-Faso^74^. Given that IgM might contribute to naturally acquired immunity against malaria^79–85^, we investigated the reactivity and functionality of the IgM antibodies. As seen in Fig. 5A, purified IgM antibodies from vaccinees (Supplementary Fig. 5) collected at day 85 recognized native MSP1 on the surface of fixed nascent merozoites present in schizonts, as shown by immune fluorescence assays. Furthermore, purified IgM antibodies recognized numerous epitopes spread across the entire MSP1 protein, as shown using an MSP1 peptide chip consisting of 1706 15mer oligopeptides with a neighbor-to-neighbor overlap of 14 amino acids (Fig. 5B and Supplementary Data 4). A comparative analysis of the epitope landscapes revealed interpersonal variabilities, which, however, occurred independent of the MSP1_FL_ dose administered. Despite an overall variability, there were some domains with high signal intensities (top 5% binders) (Fig. 5C) comprising conserved and dimorphic epitopes. This included epitopes covering natural MSP1 processing sites, such as the canonical p83/p30 cleavage sites (SITQPLVAA SETTED), alternative cleavage sites for p38/p42 (VVQLQ↓NYDEE and PIFG↓SEDND) and the p33/p19 cleavage site (EGDKC↓VENPN)^36^. Comparing the IgM with the previously published IgG epitope landscape revealed common patterns but also distinctions (Fig. 5C).

### MSP1_FL_ immunization elicits multifunctional IgMs

**Fig. 6:**
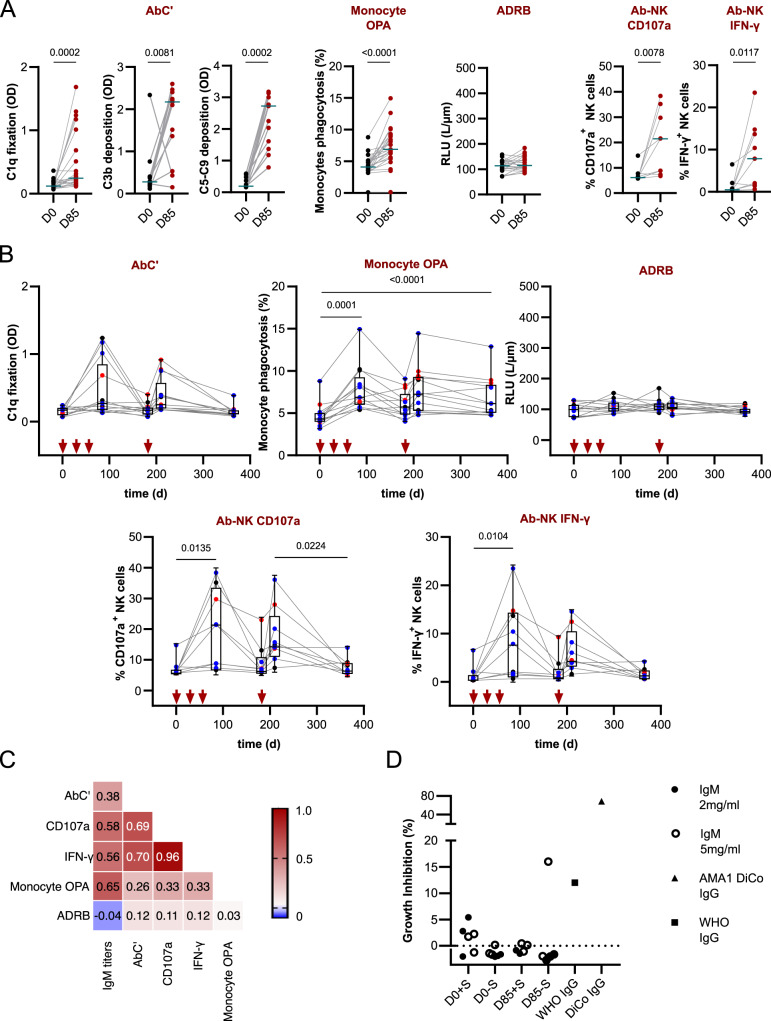
Immunization induces functional anti-MSP1_FL_ IgM. **A** Levels of Fc-mediated effector functions against MSP1_FL_ of purified IgM samples were compared between before (D0) and after full immunization (D85). Levels of antibody-dependent complement fixation (AbC’) measured by C1q fixation (*n* = 24), C3b deposition and C5-C9 deposition (membrane attack complex, MAC) (*n* = 13). Opsonic phagocytosis activity (OPA) of MSP1_FL_-coupled microsphere beads by monocytes (*n* = 24) antibody-dependent respiratory burst (ADRB) of neutrophils (*n* = 24); antibody dependent natural killer cell (Ab-NK) activity measured by degranulation (CD107a) and IFNγ production (*n* = 9). Each data point represents functional activity for one sample in duplicates. Statistical differences between timepoints were calculated using Wilcoxon test. **B** Levels of MSP1_FL_-specific Fc-mediated effector mechanisms of IgM over time. AbC‘; antibody-dependent complement fixation activity (*n* = 13). OPA; opsonic phagocytosis activity of MSP1_FL_-coupled microsphere beads monocytes (*n* = 13); ADRB); antibody-dependent respiratory burst of neutrophils (*n* = 13). Ab-NK; Fc-mediated natural killer cell degranulation, CD107a. Fc-mediated natural killer IFN-γ production; IFNγ (*n* = 9). **C** Heatmap showing spearman correlations between IgM titers and effector functions and effector functions with each other. Significant (*p* < 0.0001) spearman rank correlation coefficients for samples (*n* = 9) are highlighted in double digits. The colour intensity represents the strength of correlation. **D** Growth inhibition assay (GIA). The *P. falciparum* strain 3D7 was cultured for one cycle in the presence of total IgM from volunteers (*n* = 3) pre-vaccination (D0) and after vaccination (D85) in the presence or absence of serum and at 2 concentrations :2 mg/ml (filled circles) or 5 mg/ml (clear circles). Two IgM preparations one from MSP1-immunized rabbits (triangles) and one from semi-immune adults from Kenya (squares) served as positive controls. Each data point represents functional activity for one sample in duplicates.

Lymphocytes, NK cells and possibly monocytes^80^ express the IgM Fc-receptor, FcµR, and could potentially be stimulated by IgM from the MSP1_FL_ vaccinees. This was indeed found. Purified IgM from MSP1_FL_ vaccinees specifically stimulated phagocytosis of MSP1-coated beads by monocytes (isolated from PBMCs and used in this assay due to the absence of the FcµR in the THP1 cell line^86^) (Supplementary Fig. 6) and degranulation and IFN-γ production by NK cells in the Ab-NK assays (Fig. 6A, Supplementary Data 5). As expected, IgM were unable to activate neutrophils to release ROS in the ADRB assay. We further noted IgM-mediated activation of the classical complement pathway, as shown by C1q fixation, C3b deposition and the formation of the membrane attack complex (Fig. 6A, Supplementary Data 5). In general, the induction and decay kinetics of IgM-mediated effector functions resembled those of IgG although at a lower scale (Fig. 6B, Supplementary Fig. 7, Supplementary Table 1 and Supplementary Data 5). IgM-mediated effector functions were independent of the MSP1 dose administered to the vaccinees, but moderately correlated with the IgM titers and their time courses (Spearman rank correlation coefficients ranging from *r* = 0.65 (*p* < 0.0001) for monocytes, *r* = 0.58 for Ab-NK:CD107a*, r* = 0.56 (*p* < 0.0001) for Ab-NK: IFN-γ (*p* < 0.0001) and *r* = 0.38 (*p* = 0.01) for AbC’ (Fig. 6C, Supplementary Data 5). Varying degrees of correlation were further found between the different IgM-mediated effector functions evaluated (Fig. 6C, Supplementary Data 5). As antibodies targeting MSP1 processing sites have been associated with blocking invasion of erythrocytes by merozoites, we further tested the ability of IgM to inhibit invasion of erythrocytes by adding 2 mg ml^−1^ or 5 mg ml-1 purified IgM from MSP1_FL_ vaccinees, both with and without complement-active naïve serum, to parasite cultures for one cycle. No growth inhibitory activity (GIA) was observed, consistent with previous results using IgG from MSP1_FL_ vaccinees74. As a positive control, we used rabbit α-AMA-1 (DiCo) sera, which strongly inhibited parasite growth by 68.7%. IgG purified from a semi-immune population from Kenya inhibited parasite growth by 12% (Fig. 6D and Supplementary Data 5).

### Vaccination with MSP1_FL_ can recall a memory T cell response

As reported previously^74^, PBMCs were collected from MSP1_FL_ vaccinees at days 0 and 85 for monitoring MSP1-induced cellular immune responses. However, vaccination with MSP1_FL_ had no detectable effect on the CD4^+^, CD8^+^ T, and *γδ*T cell populations, B cell subsets (Supplementary Fig. 8, Supplementary Data 6), T follicular helper (Tfh) cells including Tfh1, Tfh2 and Tfh1–17 (Supplementary Fig. 8, Supplementary Fig. 9 and Supplementary Data 6), and different memory phenotypes (naïve, central memory, effector memory and TEMRA) of CD4^+^ and CD8^+^ T cells (Supplementary Fig. 9 and Supplementary Data 6). There was only a significant decrease in the Tfh17 subset on day 85 compared with day 0 (*p* = 0.0138) (Supplementary Fig. 8 and Supplementary Data 6). We next looked for signatures of activation that were in line with the demonstrated in vitro functionalities of the IgG and IgM antibodies from the MSP1 vaccinees.

**Fig. 7:**
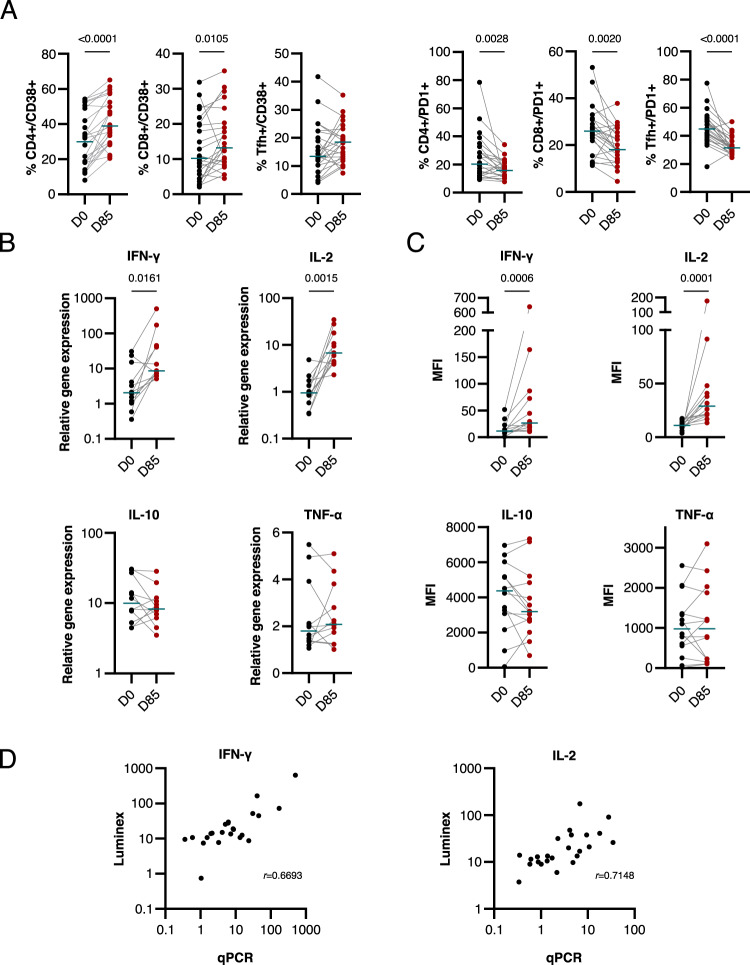
Activation of T cell-specific memory responses post-MSP1_FL_ stimulation. **A** Proportional changes of CD38^+^ T cells and PD1^+^ T cells before first (D0) and after full vaccination (D85). An increase in CD38 expression among T cells was observed after the third vaccination. This increase of CD36^+^ was significant among CD4^+^ (*p* < 0.0001) as well as CD8^+^ (*p* = 0.0105) populations. The exhaustion marker PD1^+^ decreased among all observed T cell subsets (CD4+ *p* = 0.0028; CD8+ *p* = 0.002; Tfh *p* < 0.0001). All comparisons were performed as repeated measures using Wilcoxon signed-rank test. **B** Relative gene expression of four cytokines as determined by qPCR. Relative gene expression of IFN-γ, IL-2, IL-10 and TFN-α after 16 h of stimulation with MSP-1 was determined using the ∆∆CT method using 18sRNA as reference gene and unstimulated control samples serving as background (*n* = 12). **C** Median fluorescence intensity (MFI) for cytokines IFN-γ, IL-2, IL-10 and TNF-α measured in a bead-based multiplex assay in supernatants of 48 h cultured PBMCs. Extracellular cytokine concentrations showed significant increase for IL-2 (*p* = 0.0001) and IFN-γ (*p* = 0.0006) after vaccination compared to pre- vaccination levels (*n* = 14). **D** Correlations of relative gene expression measured by qPCR and mean fluorescence intensity (MFI) of secreted cytokines IFN-γ (r = 0.67, *p* = 0.0003) and IL-2 (r = 0.71, *p* < 0.0001), Spearman correlation coefficient.

We found a significant increase in the expression level of CD38^+^ in CD4^+^ and CD8^+^ T cells (*p* < 0.0001 and *p* = 0.0105 respectively) and a trend in Tfh cells (Fig. 7A and Supplementary Data 6). CD38^+^ is a marker for cell differentiation, activation and cytotoxic potential and is associated with a reduced parasite burden^87–89^. Other markers of activation, such as ICOS, did not follow the same trend (Supplementary Fig. 8 and Supplementary Data 6). Interestingly, the proportion of PD1+ CD4+ T cells, CD8+ T cells and Tfh cells was significantly reduced on day 85 compared with day 0 (*p* = 0.0028, *p* = 0.0020 and *p* < 0.0001 respectively) (Fig. 7A and Supplementary Data 6). PD1 is a marker of exhaustion^90,91^. We further investigated the expression of 38 cytokines, using a bead-based multiplex assay, but observed no alterations between PBMCs collected from MSP1_FL_ vaccinees on day 0 and 85 (Supplementary Fig. 10 and Supplementary Data 6). This finding suggests that the CD38^+^/PD1^−^ profile is induced by the MSP1_FL_ vaccination and not the result of a concomitant inflammation or infection.

To investigate the recall T cell memory responses after MSP_FL_ vaccination, we investigated the differential gene expression profiles of the cytokines, IFN-γ, TNF-α, IL-2 and IL-10, after re-stimulation with MSP1_FL_ for 16 h. PBMCs collected at day 85 responded to an MSP1_FL_ stimulus with a significant increase in the expression levels of IFN-γ and IL-2 (*p* = 0.0161 and *p* = 0.015 respectively) but not TNF-α and IL-10 (Fig. 7B, Supplementary Fig. 11 and Supplementary Data 6), as demonstrated by transcript-specific real-time RT-PCR. Consistent with a higher transcriptional activity, the supernatants from the MSP1_FL_ stimulated PBMCs for 48 h hrs contained significantly increased amounts of IFN-γ and IL-2 (*p* = 0.0006 and *p* = 0.0001 respectively) (Fig. 7C and Supplementary Data 6). In comparison, levels of TNF-α and IL-10 did not vary. In general, the expression levels and the amount of secreted cytokines correlated (*r* = 0.67 for IFN-γ, *r* = 0.50 for TNF-α and *r* = 0.71 for IL-2; *p* = 0,0003, *p* = 0,00121 and *p* < 0.0001, respectively) (Fig. 7D, Supplementary Fig. 11, Supplementary Data 6). These finding are consistent with a recall memory that when activated results in the release of IFN-γ and the T cell specific IL-2, two factors known to contribute to parasite elimination.

### Vaccination with MSP1_FL_ induces antigen-specific IFN-γ production by CD8^+^ T cells

**Fig. 8:**
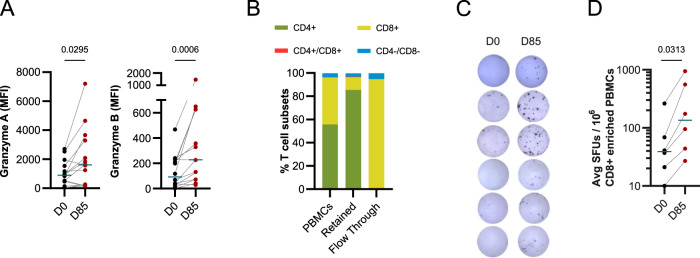
MSP1-specific recall of CD8^+^ T cell response and secretion of cytotoxic Granzyme A and B. **A** Median fluorescence intensity (MFI) in the bead-based multiplex assay for cytokines Granzyme A and Granzyme B measured in supernatants of 48 h cultured PBMCs in presence of MSP-1 showing significant increases in Granzyme A and Granzyme B levels after vaccination compared to pre-vaccination levels (*n* = 14) (Pairwise comparison using Wilcoxon signed-rank test). **B**–**D** CD8^+^ enriched PBMCs from six volunteers immunized three times with *SumayaVac- 1* were able to show recall T cell response as defined by IFN-γ production in ELISpot. **B** Subpopulations of CD4^+^ and CD8^+^ in the sample before enrichment as well as in retained and flow through fractions after magnetic beads-based enrichment of CD8^+^ T cells from PBMCs from vaccines. **C** Representative images of ELISpot wells are shown for six different vaccinees at D0 and D85. **D** CD8^+^ enriched samples (*n* = 6) showing IFN-γ secretion in the ELISpot assay upon stimulation with MSP1_FL_. The averaged spot-forming units (SFUs) per 1 million PBMCs were calculated and compared between time before first and after the third immunization showing significant increase (*p* = 0.0313). Each data point represents the mean of triplicates after subtraction of unstimulated controls normalized by the number of cells counted per well. Paired measurements were assessed using Wilcoxon signed-rank test.

We have previously attributed the recall memory to CD4^+^ T cells since the GLA-SE adjuvant included in the vaccine is known to stimulate Th1 CD4^+^ T cell responses to co-administered antigens^74^. However, the cultured IFN‐γ ELISpot assay used in the previous study relied on total PBMC populations and did not differentiate between CD4^+^ and CD8^+^ T cells, both of which are able to secrete IFN‐γ upon stimulation. In order to disentangle the role of CD8^+^ T cells in IFN‐γ production, we initially measured the levels of secreted Granzyme A and B in PBMC supernatants of stimulated cultures, using a bead-based multiplex assay, as these two cytokines are mainly produced by CD8^+^ T cells and NK cells^92^, and to a lesser extent by CD4^+^ T cells^93^. As seen in Fig. 8A, there was a significant rise in Granzyme A and B levels at day 85 compared with day 0 (*p* = 0.0296 and *p* = 0.0006 respectively) (Fig. 8A and Supplementary Data 7). This finding prompted us to sort magnetically CD8+ T cells from the PBMC collected from the MSP1_FL_ vaccinees pre-and post-vaccination (days 0 and 85), yielding an enrichment of >95% (Fig. 8B, Supplementary Fig. 12 and Supplementary Data 7). The recall capability of the purified CD8^+^ T cells were subsequently investigated in the cultured IFN‐γ ELISpot assay. We found significant increases in the production of spot forming units (SFU) between day 0 and day 85 upon stimulation with MSP1_FL_ (*p* = 0.0312) (Fig. 8C, D and Supplementary Data 7). This finding suggests that vaccination with MSP1_FL_ can also elicit a CD8+ T cell response that is responsive to a recall stimulus and, thus, may indicate a CD8^+^ T cell memory specific to MSP1.

## Discussion

The recently conducted first-in-human clinical trial of MSP1_FL_, formulated with GLA-SE as adjuvant, offered a reassessment of the vaccine potential of this malaria antigen^74^. The MSP1_FL_^−^-based vaccine was safe and immunogenic, with all vaccinees seroconverting and producing long-lasting high IgG and IgM antibody titers. Here, we show that both IgG and IgM antibodies are functional and can stimulate several Fc-mediated immune effector mechanisms associated with a reduced parasite burden and, possibly, protection. Furthermore, vaccination with MSP1_FL_ elicited recallable memory CD8^+^ T cell responses.

Several previous studies have highlighted the antiparasitic potential of antibodies targeting MSP1 by demonstrating that such antibodies can block merozoites from invading erythrocytes in a Fab-mediated manner^37^. This blocking activity is particularly evident for antibodies targeting interaction domains and natural processing sites, thereby preventing the formation of the MSP1 complex and neutralizing its role in host cell invasion and egress^13,40,44^. Although vaccination with MSP1_FL_ elicited a broad humoral immune response across the entire protein, as demonstrated by fine-scale epitope mapping, the produced IgG and IgM antibodies displayed no neutralizing activity in the growth inhibition assay. This finding seemingly contrasts with the results of our epitope mapping showing antibody binding to epitopes close to and/or covering processing sites. We speculate that the lack of GIA activity might be explained by a competition of neutralizing with interfering antibodies^15^. Alternatively, levels of MSP1-specific IgG and IgM antibodies may be insufficient for GIA activity as high levels are required to obtain growth inhibition^20^.

The inertness in GIA activity contrasts with the ability of MSP1_FL_-induced antibodies to stimulate several branches of the Fc-mediated innate immune response. This included, monocytes, neutrophils and NK cells and involved effector mechanisms, such as phagocytosis, the release of reactive oxygen species and the production of IFN-γ. In addition, MSP1_FL_-induced antibodies were able to activate the classical complement pathway, including formation of the membrane attack complex. Antibody-mediated activation of effector cells can affect parasite invasion and development in vitro^29,94,95^, and is highly correlated with protection against malaria^22–24,26,29,96^, with the breadth of Fc-mediated effector functions against merozoites delineating the grade of clinical manifestation and parasite replication^32^. Similarly, complement can impair the viability of blood stage parasites and is also significantly associated with protection against clinical malaria^22,23,97^. We consider it a quality feature of the MSP1_FL_ vaccination strategy to elicit broadly functional antibodies at titers sufficient to stimulate several immune mechanisms associated with controlling blood stage development of *P. falciparum*; yet, whether this will be sufficient needs further study. It is noteworthy that this conclusion is valid for both MSP1-induced IgG and IgM antibodies, albeit the IgM-stimulated responses were generally lower than those of IgGs.

IgM antibodies trigger effector cells via binding to the specialized Fc-receptor, FcµR, initially discovered on B-, T-, and NK cells^81^. Accordingly, IgM antibodies from MSP1_FL_ vaccinees were able to activate NK cells to produce IFN-γ and degranulate. In contrast, IgMs failed to stimulate neutrophils in the ADRB assay, which is explained by FcµR being absent in these cells^98^. On the other hand, monocytes mediated phagocytosis of opsonized MSP1_FL_-coated microbeads. Although monocytes were initially classified devoid of FcµR, recent findings suggest that human primary monocytes can express FcµR, depending upon their activation state (*67*). Furthermore, monocytes have recently been shown to phagocytose IgM-opsonized merozoites in the OPA assay^80^. Our finding that MSP1_FL_ elicited relevant IgM-mediated immune responses, supports previous reports implicating IgM antibodies in contributing to protection against malaria and being at the forefront of the defense against a reinfection^85^. The reported longevity of the MSP1_FL_-induced IgM antibodies resembling the long half-life of IgMs in malaria-exposed people^80^, raises further hopes of IgM antibodies contributing to the putative efficacy of the MSP1_FL_-based vaccine.

Previous clinical trials evaluating the safety and efficacy of MSP1 have focused on the C-terminal p42 subunit or composites of short MSP1 domains^63–66^, while ignoring the large N-terminal p83 subunit. The p83 subunit plays a pivotal role in the structure and function of MSP1. It participates in the wing domain, which contains major interaction domains for binding of various erythrocyte factors and other merozoite surface proteins^47^. Furthermore, the p83 subunits harbors the highly conserved blocks 1, 3 and 5 that are crucial for a strain-transcending immunity^11^. As shown in this and our previous study^74^, the p83 subunit is highly immunogenic and contains multiple IgG and IgM B cell epitopes. Anti-p83 antibodies are major contributors to immunogenicity^37,99,100^ and, as shown in this study, stimulated various effector cells. In addition, MSP1_FL_-induced anti-p83 antibodies confer a strain-transcending activity^11^, as previously demonstrated^74^. The cross-strain variability of the Fc-mediated effector functions triggered by *SumayaVac-1* was also demonstrated by using both, MSP1-D and MSP1-F prototypic sequences.

We have recently hypothesized that the MSP1_FL_ vaccine is able to elicit a memory T cell response. This assumption was based on a positive cultured ELISpot assay in which PBMCs collected from vaccinees more than 4 months after the last vaccination responded to a recall stimulus with the release of IFN-γ^74^. It was assumed at the time that the responding cells were of CD4^+^ type given the history of the GLA-SE adjuvant included in our vaccine as a stimulator of Th1 CD4^+^ T cells. Our current findings draw a more nuanced picture and, in addition to CD4^+^ T cells, point towards CD8^+^ T cells as also contributing to the recall memory. Firstly, PBMCs collected from vaccines on day 85 secreted high levels of Granzyme A and B upon stimulation. These two cytokines are usually produced by CD8^+^ T cells and NK cells^92^, and to a lesser extent by CD4^+^ T cells^93^. Secondly, PBMC preparations depleted of CD4^+^ T cells and CD56^+^ NK cells and, hence, enriched for CD8+ T cells responded to a recall stimulus with MSP1_FL_ with an increased production of IFN-γ. While this finding provides initial evidence for a CD8^+^ recall memory, we cannot rule out the possibility of some of the IFN-γ being produced by a CD8^+^ NK cell subpopulation^101^ or remnant CD56^dim^ NK cells (Supplementary Fig. 12). However, we do not think that NK cells significantly contributed to IFN-γ release since the main contributor to IFN-γ production in NK cells are the CD56^bright^ NK cells^102^ subpopulation that was efficiently removed from the CD8^+^-enriched fractions (Supplementary Fig. 12). Regardless, we acknowledge the need for further studies evaluating the potential of the MSP1_FL_ vaccine to elicit a CD4+ and CD8+ T cell recall memory including its breadth and longevity. An MSP1_FL_-induced CD8+ T cell-mediated cytotoxicity might expand the efficacy of the vaccine towards liver stages, given that some studies have associated the protective immunity elicited by the liver stage vaccine, RTS,S, with a CD8^+^ T cell response^103,104^.

In summary, our finding that *SumayaVac-1* elicits broad, functional B and T cell responses implicated in controlling a *P. falciparum* infection at the blood, revitalizes MSP1 as a relevant malaria vaccine candidate. Studies are currently ongoing in a phase I conducted in Tanzania to evaluate further the efficacy, breadth and longevity of the MSP1_FL_ induced immune responses in a pre-exposed population. A CHMI challenge will complement this trial as proof of efficacy through protection after challenge or delay in time to patency and/or asexual blood stage growth rates.

## Methods

### Ethical approval

The trial was performed following the principles of good clinical practice, in accordance with the ethical principles described in the Declaration of Helsinki (6th revision, 2008) and registered with EudraCT (No. 2016-002463-33; date of first approval 31 January 2018). The protocol and amendments were approved by the Ethics Committee of the Medical Faculty of Heidelberg (Ethical vote AFmo-538/2016) and the relevant regulatory authority (Paul Ehrlich Institute, Langen, Germany). All volunteers were fully informed about the trial and gave their written consent. A single-center, randomized, double-blind, placebo and adjuvant-controlled phase Ia first-in-human dose escalation trial was conducted at the ISO-certified clinical trial unit of the Clinical Pharmacology and Pharmacoepidemiology Department of the Heidelberg University Hospital. Sixteen participants were included in two consecutive cohorts, each randomizing 12 volunteers into a vaccine + adjuvant group, and two participants each receiving placebo or adjuvant only. The safety of the study and any dose increase was supervised by an independent Data and Safety Monitoring Board (DSMB). Further information regarding the phase 1a can be found in Blank & Fürle et al., 2020^74^. The clinical trial protocol and the ethical votes are available at DRYAD 10.5061/dryad.kwh70rz0f.

### Samples

The clinical trial blood samples were collected from the participants during the trial at the mentioned time points using S-Monovette serum-gel vacutainers and a clotting activator (Sarstedt). The clotted blood was centrifuged for 10 min at 2500 × *g* and obtained serum was stored at −80 °C^74^. To obtain an anti-malaria human serum WHO reference reagent for the experiments, serum from individuals living in Kimusu, Kenya, was pooled by the National Institute for Biological Standards and Control (NIBSC code 10/198). The individuals have been exposed to malaria in the past^74^.

### IgG and IgM purification of clinical trial serum samples

IgG was purified from sera by protein-G affinity chromatography (Pierce, Thermo Fisher Scientific) according to the manufacturer`s instructions. IgM was purified from sera with CDI (Carbonyldiimidazole)-activated agarose (Thermo Fisher Scientific, capture select antibody affinity resins) according to the manufacturer`s instructions. The IgG/IgM eluate was sterile filtered (0.22 µm), dialyzed into RPMI 1640 and concentrated with Amicon ultra centrifugal filters (Millipore). The IgG/IgM concentration was adjusted to 15 mg/ml and 5 mg/ml in RPMI 1640, aliquots were stored at −20 °C.

### Culture of THP1 cells

THP1 cells (KEMRI-Wellcome Trust Research Programme) were maintained in RPMI 1640 medium with 2 mM L-glutamine, 10 mM HEPES, 1% pen strep (10,000 units/ml penicillin and 10,000 μg/ml streptomycin) and 10% fetal bovine serum (FBS) in a humidified incubator with 5% CO_2_ at 37 °C. Cell density was monitored daily and maintained at concentrations between 1 × 10 × 105 and 1 × 10^6^ cells/ml. Cells were passaged when cell density exceeded 1 × 10^6^ cells/ml or at day 6.

### Isolation of monocytes, NK cells and neutrophils from peripheral blood

Monocytes, natural killer (NK) cells and neutrophils were isolated from fresh human peripheral blood using established protocols^29,74^. Briefly, blood from malaria naïve donors was collected in heparin vacutainers and separated using a Histopaque (Sigma-Aldrich) gradient. The peripheral blood mononuclear cell (PBMC) and neutrophil layer were separated. For monocyte and NK cell isolation, PBMCs were washed with ice-cold culture media (RPMI 1640 media with 2 mM L- glutamine, 10% fetal calf serum (FCS) and 1% penicillin-streptomycin) and counted using a hemocytometer. Monocyte isolation and NK cell isolation from PBMCs was performed by using the Monocyte Isolation Kit (Miltenyi Biotec) or the NK cell isolation kit (Miltenyi Biotec), respectively as per manufacturer’s instructions. Monocytes and NK cells were stored in ice-cold culture media until use. Neutrophils were isolated from the erythrocytes pellet after Histopaque gradient centrifugation followed by dextran segmentation and hypotonic erythrocytes lysis. Neutrophils were stored in either ice-cold culture media or PBS for opsonic phagocytosis or ADRB assays, respectively. For opsonic phagocytosis assays, phagocytes (monocytes, neutrophils) were adjusted to 3.3 × 10^5^ phagocytes/ml. For ADRB, neutrophils were adjusted to 10 × 10^6^ cells/ml and for Ab-NK, NK cells were adjusted to 2.5 × 10^5^ NK cells/ml.

### C1q fixation ELISA assay

The MSP1 C1q fixation assay was performed according to published methods^22,74^. The plates were coated with recombinant MSP1_FL_ (MSP1-D or MSP1-F) or MSP1 subunits at 100 nM and incubated overnight at 4 °C. The next day, the plates were washed four times with 200 µl 1x PBS containing 0.05% Tween 20 (PBST) and blocked with 200 µl/well of 1% casein/PBS at 37 °C for 2 h. After washing, the plates were incubated with 50 µl of purified IgG or IgM at 1 mg/ml or 2 mg/ml in blocking buffer, respectively at 37 °C for 1 h. After incubation, the plates were washed and 40 µl/well of recombinant C1q (Merck Millipore #204876) at 10 µg/ml in blocking buffer was added for 30 min at 37 °C. To detect C1q binding, 50 µl/well of anti-C1q horse radish peroxidase (HRP)-conjugated secondary antibodies (Abcam #ab46191) was added at a 1:100 dilution in blocking buffer for 1 h at 37 °C. For signal development, 100 µl/well of SigmaFAST OPD (Sigma-Aldrich) was added for 30 min to 1 h in the dark at room temperature. The reaction was stopped with 30 µl of 1 M HCL and the signal intensity was measured at 492 nm using the Biotek Cytation 3 plate reader and the Gen 5 acquisition software.

### C3b and C5-C9 deposition ELISA assays

The C3b and C5-C9 ELISA was performed as the C1q ELISA using the MicroVue Complement iC3b EIA (Quidel #A029) and the MicroVue Complement Sc5b-9 Plus EIA kit (Quidel #A029). After coating of recombinant MSP1_FL_ (100 nM) and opsonization with IgG (1 mg/ml) and IgM (2 mg/ml), 50 µl/well of either C3b or SC5b-9 conjugate was added and incubated for 30 min at 37 °C. For signal development, 100 µl/well of conjugate specific substrate solution was added for 30 min at 37 °C and stopped with conjugate specific stop solution. Absorbance was read at 405 nm (for C3b detection) and 450 nm (for C5-C9 detection).

### Antibody-dependent respiratory burst (ADRB) assay

The MSP1_FL_-specific ADRB assay^54,105^ was performed by coating recombinant MSP1_FL_ and subunits at 100 nM in PBS in opaque 96-well Lumitrac microplates (Greiner Bio-One), blocked with 200 µl/well of sterile 1% casein/PBS and incubated for 1 h at 37 °C with 50 µ/well of IgG or IgM at 1 mg/ml or 2 mg/ml in PBS, respectively. The plates were washed with PBS and 50 µl/well of luminol at 0.04 mg/ml were added to each well. Next, 50 µl/well of freshly isolated neutrophils were added quickly at 10 × 10^6^ cells/ml and absorption was immediately read at 450 nm using the Biotek Cytation 3 reader for every 2 min over 1 h.

### Coupling of recombinant antigens to microsphere beads

The coupling of recombinant MSP1_FL_ (MSP1-D or MSP1-F) and subunits^77^ was performed using 25 µg of recombinant antigens that were coupled to fluorescent microsphere beads (Polyscience #18660) of 1 µm size in borate buffer overnight at room temperature in the dark while gently shaking. Beads were centrifuged at 2000x g for 7 min and washed thrice in blocking buffer (10 mg/ml BSA in borate buffer) for 30 min at room temperature. After blocking, the beads were adjusted to 1.5 × 10^9^ beads/ml in ice-cold PBS supplemented with 5% glycerol and 0.1% sodium azide and stored at 4 °C.

### Opsonic phagocytosis activity assays

Opsonic phagocytosis activity (OPA) assays using either MSP1_FL_ (MSP1-D or MSP1-F) or subunits coupled to fluorescent beads were performed according to a published protocol^77^ using different phagocytes (THP1 cells, isolated monocytes and neutrophils). Briefly, 50 µl/well of antigen coupled bead suspension (containing 7.5 × 10^6^ beads) was added to 96-well to U-bottomed plates and opsonized for 1 h at 37 °C with 50 µl/well of purified IgG or IgM at 12.5 µg/ml or 25 µg/ml, respectively. The plates were centrifuged at 2000 × *g* for 7 min and washed thrice with 200 µl/well PBS to remove unbound antibodies. Opsonized beads were resuspended in 50 µl/well culture medium (RPMI 1640 media with 2 mM L-glutamine, 10% FCS and 1% penicillin-streptomycin) before incubation with 5 × 10^4^ phagocytes in 200 µl/well final volume for 30 min at 37 °C. Phagocytosis was arrested by centrifugation at 1200 rpm for 7 min at 4 °C and washing with 200 µl/well ice-cold FACS buffer (0.5% BSA and 2 mM EDTA in PBS). Cells were resuspended in 200 µl/well ice-cold 2% formaldehyde/PBS and the proportion of phagocytes containing PE fluorescent beads in the P2 channel was measured by flow cytometry using the FACS Canto II (BD biosciences). An example of the gating strategy can be found in Supplementary Fig. 6. Data analysis was performed with the FlowJo V10 software.

### Ab-NK activity assay

The Ab-NK activity assay was performed according to the recently published protocol^29^ with modifications. Briefly, 100 nM of MSP1_FL_ was coated on 96-well plates overnight at 4 °C. The plates were washed thrice with 200 µl/well sterile PBS and blocked with sterile 1% casein/PBS for 4 h at 37 °C. The plates were washed and incubated for 2 h at 37 °C with IgG and IgM at 1 mg/ml or 2 mg/ml, respectively. After incubation and washing, 5 × 10^4^ freshly isolated human NK cells together with anti-human CD107a PE (BD biosciences #560948, 1:70), brefeldin A (Sigma-Aldrich #B6542, 1:200) and monensin (Sigma #M5273, 1:200) was added for 18 h at 37 °C. Afterwards, the cells were transferred to 96-well V-bottomed plates, centrifuged at 300 × *g* for min at 4 °C and washed with ice-cold FACS buffer (PBS with 1% BSA and 0.1% sodium azide). Cell viability was determined by adding 10 ul/well of fixable viability dye eFluor^TM^520 (Thermo Fischer) for 10 min at 4 °C. NK cell surface markers were labelled with 20 µl/well of an antibody cocktail of anti-CD56 APC (BD biosciences #555518, 1:17) and anti-CD3 PE-Cy5 (BD biosciences #561007, 1:33) for 30 min at 4 °C in the dark. After washing, NK cells were fixed with 80 µl/well of CellFIX (BD biosciences) for 10 min at 4 °C and permeabilized with 80 µl/well of permwash (BD biosciences) for 10 min at 4 °C. Intracellular IFN-γ was measured by adding 30 µl/well of anti- IFN-γ PE-Cy7 (BD biosciences #560924, 1:33) for 1 h at 4 °C. NK cells were washed with permwash, resuspended in 150 µl/well FACS buffer and Ab-NK activity (proportion of NK cells with CD107a and/or IFN-γ staining was assessed by flow cytometry (FACS ContoII, BD biosciences) and analysed by the FlowJo V10 software.

### *P. falciparum* in vitro culture and synchronization

*P. falciparum* 3D7 were cultured in 12 ml malaria culture medium (Cmed: RPMI, 20% serum, Hypoxanthine, Gentamycin) in 25 cm^2^ culture flasks at hematocrit of 4% using human 0+ erythrocytes. They were kept in an atmosphere of 5% CO_2_, 5% O_2_, 90% N2 at 37 °C. To assess the level of parasitemia, 0.5 ml of the culture was centrifuged at 300 g, 3 min. and the supernatant was removed. A drop of blood was applied on a glass slide and smeared using another slide. The smear was fixated and stained using Giemsa staining kit RAL 555 (Ral Diagnostics). At least 300 uninfected erythrocytes and the different infected erythrocytes were counted under the microscope to determine parasitemia and the different parasite stages:

$$Parasitemia=\frac{infected\,erythrocytes}{total\,erythrocytes}\ast 100$$

### *P. falciparum* growth inhibition assay (GIA)

A synchronous *P. falciparum* 3D7 culture at schizont stages was adjusted to 0.6% parasitemia in 4% hematocrit using Cmed, and also Cmed without human serum to examine the influence of active complement on the inhibition process. The prediluted antibodies were added to 25 µl/well parasite suspension in Cmed with or without human serum to achieve a final IgM concentration of 2 mg/ml and 5 mg/ml per well. Samples were pipetted in duplicates in 96-well U-bottom culture plates with individual lids. Rabbit IgG against AMA-1 (DiCo, BioGenes Germany #BG98) (purified IgG obtained from rabbits immunized with a mixture of 7 AMA-1 alleles and purified at BioGenes, Berlin, Germany) and WHO standards (NIBSC, #10/198) were used as positive control and plain erythrocytes as negative control. As parasite control, 25 µl RPMI and 25 µl parasite suspension were pipetted in four wells without antibodies. The plates were incubated at 37 °C in a gas chamber containing 5% CO_2_, 5% O_2_, 90% N_2_ for one parasite cycle (40–48 h). Each well was resuspended with cold PBS and the plates were frozen at −20 °C to lyse the cells. After thawing, each well was resuspended with *p*LDH (*plasmodium* Lactate DeHydrogenase) substrate buffer containing NBT-tablet (2 mg/10 ml), 50 μl APAD (stock 10 mg/ml) + 200 μl Diaphorase (stock 50 units/ml) for parasite quantification by biochemical detection of *p*LDH. OD was measured at wavelength of 650 nm using Cytation 3 microplate reader. The inhibition was calculated using the following formula:

$$Inhibition[ \% ]=100-\frac{(A650\,IgG\,or\,IgM\,sample-A650\,ery\,control)}{(A650\,parasite\,control-A650\,ery\,control)}\times 100 \%$$

### Western blot

Serum, flow-through and purified IgM and IgG samples from different days after vaccination were diluted in SDS loading buffer with DTT and loaded onto 10% SDS gels. Prestained marker (NEB P7719 10–250 kDa) served as reference. BCIP was used as substrate for antibody-coupled alkaline phosphatase. Antibodies were detected using Anti-Human IgG −Alkaline Phosphatase antibody produced in goat was added (Sigma Aldrich) at 1:30 000 dilution in blocking buffer.

### IFA

Parasite cultures were synchronized using 5% sorbitol. Late staged (40–48 h post invasion) were treated with 10μm E64 for 4–6 h, then fixed in 4% paraformaldehyde in PBS for 30 min at RT while shaking. Cells were permeabilized with 125 mM gylcine/0.5% Triton-X-100/PBS for 30 min at RT, washed with PBS and blocked using 3% BSA/PBS for 1 h at RT, followed by an overnight incubation with IgM purified from serum from vaccinees (1 mg/ml in blocking buffer) at 4 °C, while gently agitating the tube the entire time. The next day, the parasites were washed thoroughly with PBS, incubated with Goat anti-human Immunoglobulins (polyvalent G, A, M) FITC (Sigma Aldrich #F6506) for 1 h followed by three washing steps, the last one with the addition of Hoechst 33342 (1 μg ml^−1^, Thermo Fisher Scientific #62249). Samples were transferred onto concanavalin A (500 μg ml^−1^, Sigma-Aldrich)-coated slides and observed using a Nikon Eclipse Ti microscope (Nikon Instruments Europe B.V.) equipped with an Orca Flash 4.0 camera (Hamamatsu Photonics K.K.).

### MSP1 epitope mapping

PEPperPRINT GmbH Heidelberg conducted high-resolution epitope mapping as reported in^74^. Briefly, MSP1 sequence (3D7) translated into linear peptides consisting of 15 amino acids with a peptide–peptide overlap of 14 amino acids resulted in a peptide microarrays contained 1706 different peptides printed in duplicate. Incubation of peptide microarray with the serum samples from vaccinees (*n* = 24) after immunization together with placebos (*n* = 4) (1:1000) was followed by staining with labeled secondary antibody for IgM. Read-out was done with a LICOR Odyssey Imaging System. As each peptide on the peptide array is printed as duplicate, the raw peptide spot fluorescence intensity is the average of the corresponding two spot duplicates.

### PBMC stimulation

Cryopreserved PBMCs sample were thawed in 1 ml warm PBMC medium (RPMI 1640, 10% fetal bovine serum, 200 mM L-glutamine, 1% penicillin/streptomycin) and supplemented with 50 U/ml Benzonase (Novagen Millipore GmbH). After washing, the cells were resuspended in warm PBMC medium, adjusted to 10^6^ PBMCs/ml and allowed to rest for at least 2 h at 37 °C (5% CO_2_, 5% O_2_, 90% N_2_). Cell numbers were determined by trypan blue staining and adjusted to 106 cells/ml. The stimulation was started by adding either full length MSP1 (final concentration of 25 µg/ml), anti-CD3 antibody as stimulation control (Mabtech #3605–1–50, final concentration 0.1 µg/ml) and PBMC medium for the unstimulated condition. For qPCR analysis, cells were collected and lysed after 16 h of stimulation. Supernatants for multiplex cytokine analysis were collected after 48 h of stimulation with MSP-1 and stored at −20 °C until used.

### Real-time PCR

RNA extraction was carried out using the RNeasy Mini-Kit (Qiagen, Crawley, UK) according to the manufacturer’s instructions. 1 million cells per condition and timepoint were pelleted by centrifugation for 5 min at 300 × *g*. After removing the supernatant, cells were lysed with 350 µl RLT buffer. Protocol was followed with optional on-column DNase digestion (RNase free DNase set, Qiagen, Crawley, UK). RNA was eluted in 30 µl RNAse free water and verified for purity and quantity using the NanoDrop™ Spectrophotometer (ThermoFisher Scientific). Up to 1 µg RNA was reverse transcribed using Omniscript RT Kit (Qiagen), 0,5 µg Oligo(dT) pimers (ThermoFisher Scientific) and 0,25 µM Random Nonamers (Sigma) per 20 µl assay, as well as RNase Inhibitor (RiboLock RNase Inhibitor (40 U/µl), ThermoFisher Scientific, Waltham, USA) at a final concentration of 0,5 U/µl. Before adding the Master Mix, RNA was denatured for 5 min at 65 °C and placed on ice until final incubation with the Master Mix for 60 min at 37 °C. qPCR was performed using the TaqMan^TM^ Fast Advanced Master Mix (ThermoFisher Scientific, Waltham, USA) according to manufacturer’s instructions using 2 µl of the cDNA template for an end volume of 20 µl. Assays for IFNγ (Assay ID: Hs00989291_m1), TNF-α (Assay ID: Hs00174128_m1), IL-2 (Assay ID: Hs00174114_m1), IL-10 (Assay ID: Hs00961622_m1) and the reference gene 18 S rRNA (Assay ID: Hs99999901_s1) were performed in technical duplicates. cDNA for the 18 S rRNA was diluted before adding to the assay 20-fold, according to 0.1 µl of cDNA. NCT and no-RT-controls were absolved on each plate and each RNA extract, respectively. Using a LightCycler^R^ 480 II (Roche Diagnostics), qPCR program (UNG activation at 50 °C for 2 min, polymerase activation at 95 °C for 20 s, 40 cycles of denaturing at 95 °C for 3 s and annealing at 60 °C for 30 s) was carried out, while fluorescence measurement took place after every cycle. Cp calculation was performed with the Abs Quant/2nd Derivate Analysis from the LightCycler^R^ Software Version 1.5. Relative quantification was done using the Pfaffl method^106^, including the (previous determined) efficiency correction for each assay. Several reference genes were evaluated to identify the best suited one for the application resulting in the selection of 18 s RNA.

### Evaluation of the reference gene

A first selection of suited reference genes for PBMCs was done by searching in the literature^107–109^. A small selection was made based on good results for stability in large comparative studies, as well as on frequently used reference genes in previous work that methodically investigate the same as we do^110–114^. Expression stability was tested according to^107^ normalized to the cell number in PBMCs from four different donors. PBMCs were isolated using the histopaque density gradient, frozen, short time stored in liquid nitrogen and then thawed. After two hours resting in the incubator, cells were counted using a Neubauer hemacytometer and adjusted to 3.33 × 10^6^ cells/ml and split into 3 Greiner tubes. To compare the unstimulated gene expression with the stimulated, cells in one tube were treated 48 h with 0.1 µg/ml final concentrated anti-CD3 and in the second tube 12 h with 1 ng/ml PMA and 1 µg/ml Ionomycin. The third tube acted as the unstimulated control to compare with the treatment and was incubated for 48 h. Right before RNA extraction, PBMCs were resuspended and carefully counted in triplicate random samples in hemocytometer by tryptan blue staining. As exact as possible, one million cells from each donor and each condition were lysed and followed by RNA extraction. From one donor only 0.5 × 10^6^ cells were taken from each condition. cDNA translation took place without adjusting the RNA amount, because of variation due to the stimulation process, but controlling that the total RNA amount is still in the optimal range of the assay. Assays for 18 S rRNA (Assay ID: Hs99999901_s1), HPRT (Assay ID: Hs99999909_m1) and GAPDH (Assay ID: Hs99999905_m1) were performed with 1 µl of the cDNA product in technical duplicates. Raw Cp values were, after averaging the technical duplicated, analysed regarding the delta Cp of the treated (anti-CD3 or PMA/Ionomycin) samples and the untreated sample and the resulting expression ratio, respectively. Because normalization took place against the cell amount, low ΔCps and expression ratios close to one indicate stable expression. More information in Supplementary Fig. 13 and Supplementary Fig. 14.

### Immunophenotyping

The phenotyping of T- and B-lymphocytes was performed in whole blood within 24 h. Absolute cell count was measured using the BD Multitest ^TM^ 6-color TBNK reagent with BD Trucount^TM^ tubes (BD Biosciences, Heidelberg, Germany). Employing comprehensive T-cell, B-cell panels, the dynamics of lymphocyte subpopulations were monitored. The panels are modified recommendations of the “Human Immunophenotyping consortium” (Maecker, H.T. et al., Nat Rev Immunol, 2012. 12(3): p. 191–200). Experimets were performed at the Institute for Immunology, Heidelberg University Hospital and German Center for Infection Research (DZIF), Heidelberg, Germany.

### CD8 IFN-γ Elispot

The human *IFN-γ* Elispot kit from Mabtech was used to detect *IFN-γ* secreting cells in PBMC samples after MSP1_FL_ antigen stimulation. Cryopreserved PBMCs were thawed and rested before returning them to culture in RPMI1640 with 10% FBS, 1% penicillin/streptomycin and L-Glutamin and adjusting cell numbers to 10^6^ PBMCs/ml. For pre-stimulation, 3 × 10^6^ cells and 106 cells were incubated for 22–24 h (37 °C; 5% O_2_, 5% CO_2_, 90% N_2_) in culture medium supplemented with MSP1_FL_ (25 µg/ml final concentration) and medium without additive, respectively. Polystyrene beads (Fluoresbrite® Microspheres 1 µm) were coated with MSP1_FL_ and stored in PBS. On the second day, MSP1_FL_-stimulated PBMC samples were depleted of CD4+ and CD56^high^ expressing cells by negative selection using magnetic beads (Miltenyi) and LS columns. Negatively selected cells, enriched in CD8+ cells, were then plated at approximately 200 000 cells/well before adding 50 µl of MSP1_FL_ coated beads (approximately 2 × x106 beads) to each well. Uncoated beads were also used as negative control. Unstimulated controls were also incubated in non-supplemented PBMC medium. Exact cell numbers were determined after plating to allow calculation of spot forming units (SFUs) later on. The samples were incubated for 24 h at 37 °C before plate development. After dumping the cells, the plate was washed with PBS before detection antibody was added and incubated for 2 h, then again washed with PBS before adding Streptavidin-ALP for one hour. Lastly, the plate was again washed with PBS before adding BCIP/NBT substrate until spots develop and the reaction stopped by dipping the plate repeatedly in ddH_2_O. Each sample was plated in triplicates and one additional well which served as positive control by addition of anti-CD3 antibody (Mabtech #3605-1-50, 0.2 µg/ml final concentration). After drying, the plate was read using the CTL ImmunoSpot Reader and analyzed using the SmartCount™ function. Spot forming units per 10^6^ cells were calculated by subtracting sample corresponding unstimulated controls and adjusting for number of plated cells.

$$SFU\,per\,{10}^{!}\,cells=\frac{mean\,stimulated\,sample-mean\,unstimulated\,sample}{mean\,counted\,cells\ast {10}^{!}}$$

### Multiplex analysis of extracellular cytokine levels

Two different bead based multiplex assay kits were purchased to analyze the cytokine environment in the vaccinees after immunization and after restimulation of PBMCs with MSP1_FL_. The first kit (Milliplex Human Cytokine Magnetic Bead Panel 41 Plex) included 38 different cytokines was purchased from Millipore for the analysis of unaltered serum samples. This assay included serum samples at day 0 and day 85 after vaccination from 16 donors in duplicates (included cytokines comprised EGF, FGF-2, Eotaxin, TGF-α, G-CSF, Flt-3L, GM-CSF, Fractalkine, IFN-α2, *IFN-γ*, GRO, IL-10, MCP-3, IL-12P40, MDC, IL-12P70, PDGF-AA, IL-13, PDGF-AB/BB, IL-15, sCD40L, IL-17A, IL-1RA, IL-1α, IL-9, IL-1β, IL-2, IL-3, IL-4, IL-5, IL-6, IL-7, IL-8, IP-10, MCP-1, TNFα, TNFβ and VEGF). The second kit was purchased from Thermo Scientific (ProcartaPlex PPX-06) for the detection of six cytokines (IFN-γ, TNF-α, IL-2, IL-10, Granzyme A and B) in supernatant of MSP1_FL_-stimulated PBMC culture (described above) to examine memory response including undiluted samples from fourteen donors at two timepoints (day 0 and day 85 after immunization) in duplicates. Both assays were performed according to manufacturer’s protocol and read using the FlexMAP3D running xPONENT 4.2 software.

### Statistical analysis

Data analysis was performed in Prism 9.3.1(GraphPad) and R. The differences in functional activities between timepoints were assessed by Wilcoxon rank test, Kruskal-Wallis test or Friedmann test followed by Dunn’s multiple comparison test, where indicated. Pairwise correlations were calculated using nonparametric Spearman’s correlations. *p* < 0.05 was considered statistically significant.

### Reporting summary

Further information on research design is available in the Nature Research Reporting Summary linked to this article.

### Supplementary information

Supplementary Material

REPORTING SUMMARY

supplementary data file 1

supplementary data file 2

supplementary data file 3

supplementary data file 4

supplementary data file 5

supplementary data file 6

supplementary data file 7

## Data Availability

The authors declare that the data supporting the findings of this study are available within the article and its supplementary information files, or are available from the authors upon request. Requests for materials should be addressed to RTL.
