# Supplementary material for: Multifunctional IgG/IgM antibodies and cellular cytotoxicity are elicited by the full-length MSP1 SumayaVac-1 malaria vaccine
Source: NPJ Vaccines. 2023 Aug 9;8:112. doi: 10.1038/s41541-023-00701-2 (PMC10412566; doi:10.1038/s41541-023-00701-2)
Supplement: Supplementary file 1 — Supplementary Material [file 41541_2023_701_MOESM1_ESM.pdf]

Title: Multifunctional IgG/IgM antibodies and cellular cytotoxicity are elicited by the full-length MSP1 "*SumayaVac-1*" malaria vaccine in a phase I clinical trial

Authors: Micha Rosenkranz,[1†] Kristin Fürle,[1†] Julia Hibbert,[1] Anne Ulmer,[1] Arin Ali,[1] Thomas Giese,[2] Antje Blank,[3] Walter E. Haefeli,[3] Ernst Böhnlein,[4] Michael Lanzer,[1] Richard Thomson-Luque[4*]

Affiliations:

[1]Center for Infectious Diseases-Parasitology, Heidelberg University Hospital, Heidelberg, Germany.

[2]Institute for Immunology, Heidelberg University Hospital and German Center for Infection Research (DZIF), Heidelberg, Germany.

[3]Clinical Pharmacology and Pharmacoepidemiology, Heidelberg University Hospital, Heidelberg, Germany.

[4]Sumaya-Biotech GmbH & Co. KG.

†Equally contributed

[*]Corresponding author: Richard Thomson-Luque - email: thomson-luque@sumaya-biotech.com

SUPPLEMENTARY FIGURES

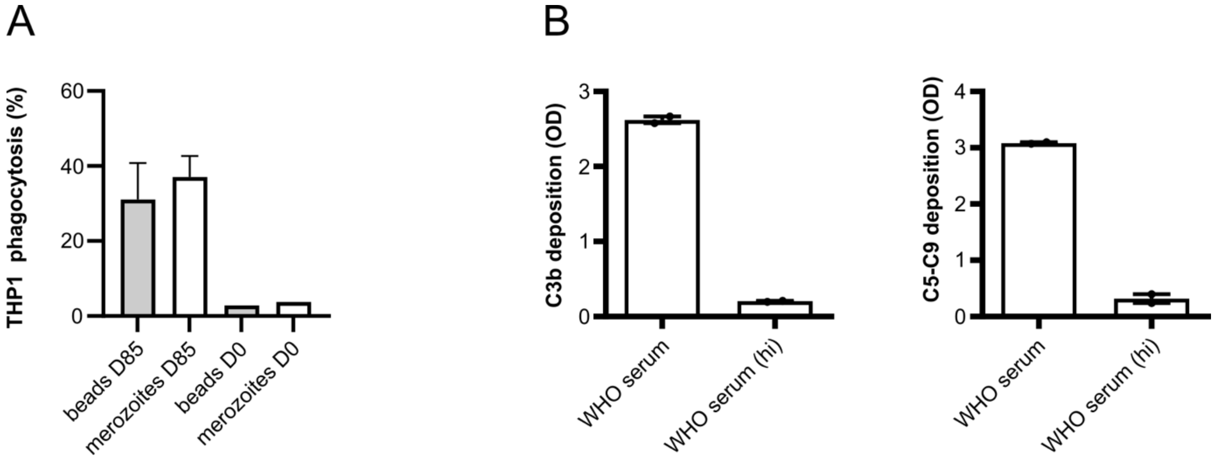

Supplementary Figure 1: Experimental controls for phagocytosis and complement fixation assays. (A) Phagocytosis of MSP1$_{FL}$-coupled beads to merozoites by THP1 cells using pre-immune and post-immune IgG. No difference between the two approaches was observed. **(B)** The role of complement in the C3b and C5-C9 deposition assays was verified with the use of heat-inactivated WHO serum (Data file S1).

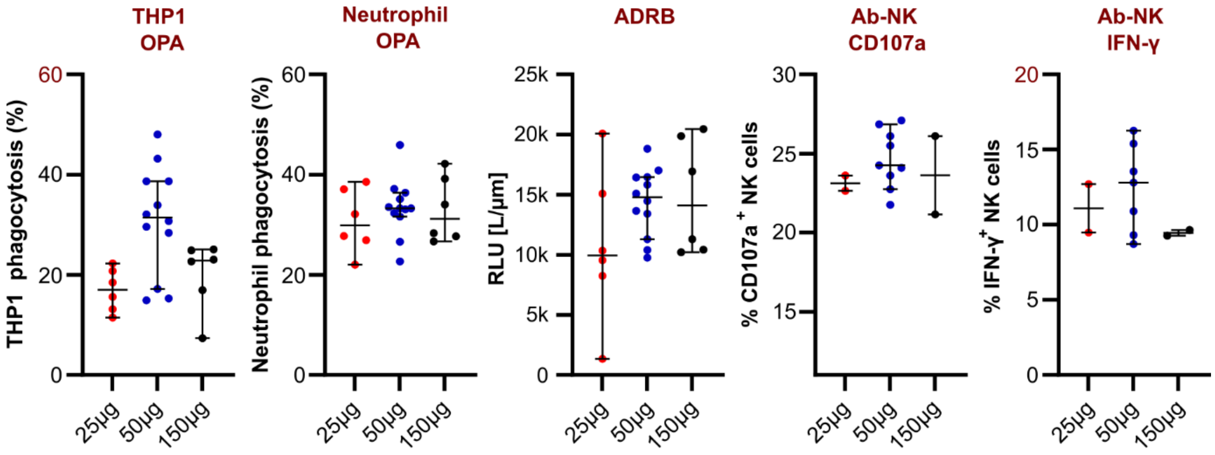

Supplementary Figure 2: Functional activity of post-immune IgG was independent of the immunization dose. Fc-mediated effector functions of post-immunization IgG (D85) was compared between the different doses (25µg; red dots, 50µg, blue dots and 150µg, black dots). Error bars represent the median plus 95% confidence intervals.

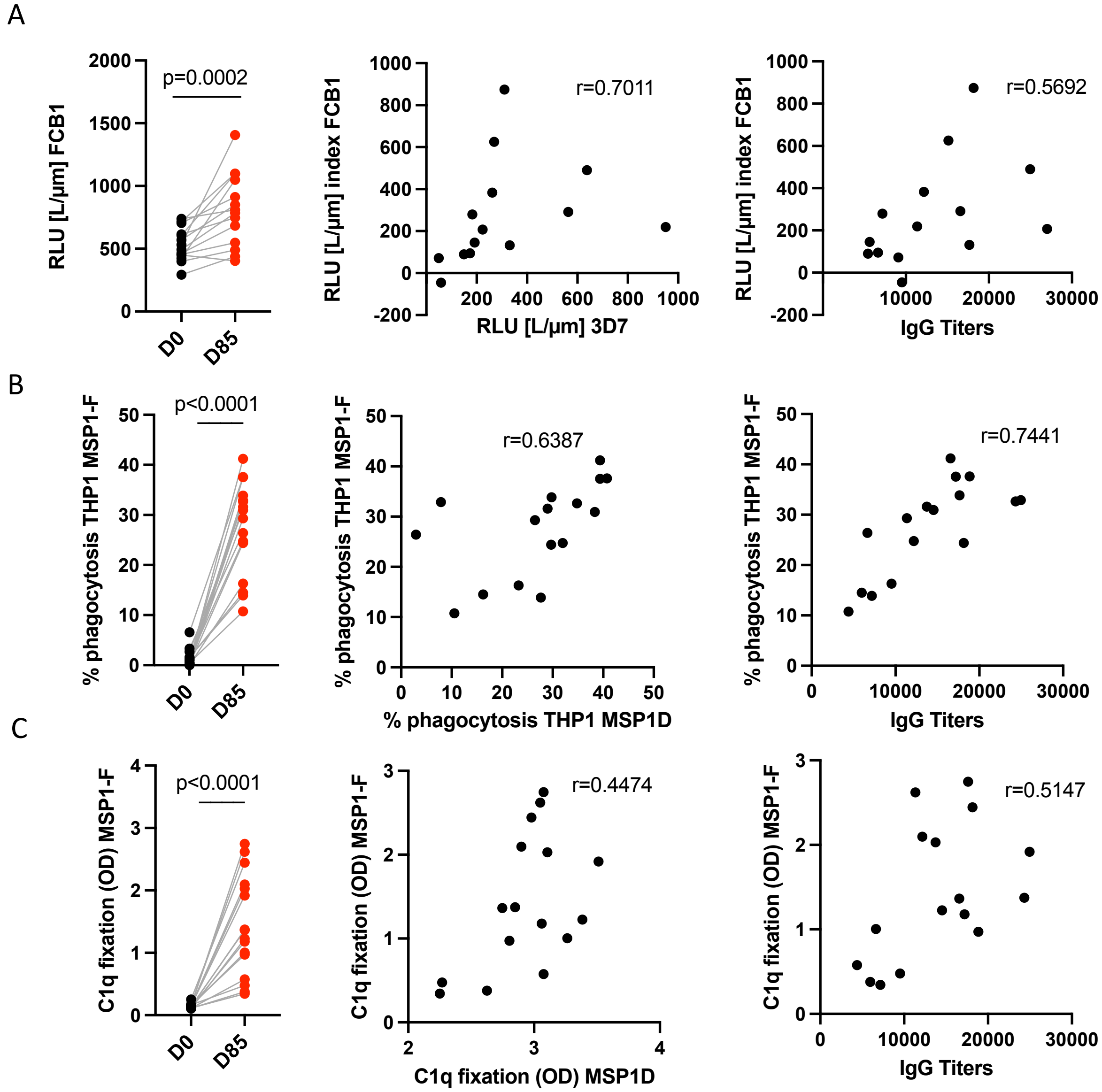

Supplementary Figure 3: Illustration of cross-strain variability of antibody-mediated effector functions. (**A**) From left to right: Significant changes in ADRB activity between D0 and D85 using the FCB1 strain (*p*=0.0002) (left). Significant correlation in ADRB activity index (corrected for the background activity at day 0 at D85 of FCB1 vs 3D7 strains (*p*=0.0067) (middle). Significant correlation in ADRB activity index of FCB1 vs IgG titers at day 85 (*p*=0.0366) (right). (**B**) From left to right: Significant changes in OPA-THP1 activity between D0 and D85 using MSP1-F (*p*<0.0001) (left). Significant correlation in OPA-THP1 activity at D85 using MSP1-F and MSP1-D (*p*=0.0091) (middle). Significant correlation OPA-THP1 activity at D85 using MSP1-F vs IgG titers at D85 (*p*=0.0014) (right). (**C**) From left to right: Significant changes in C1q fixation activity between D0 and D85 using MSP1-F(*p*<0.0001) (left). Correlation trend in C1q fixation activity at D85 using MSP1-F and MSP1-D (middle). Significant correlation C1q fixation activity at D85 using MSP1-F vs IgG titers at D85 (*p*=0.0436) (right). Statistical differences between timepoints were calculated using Wilcoxon ranked test. For correlations Spearman rank test was used.

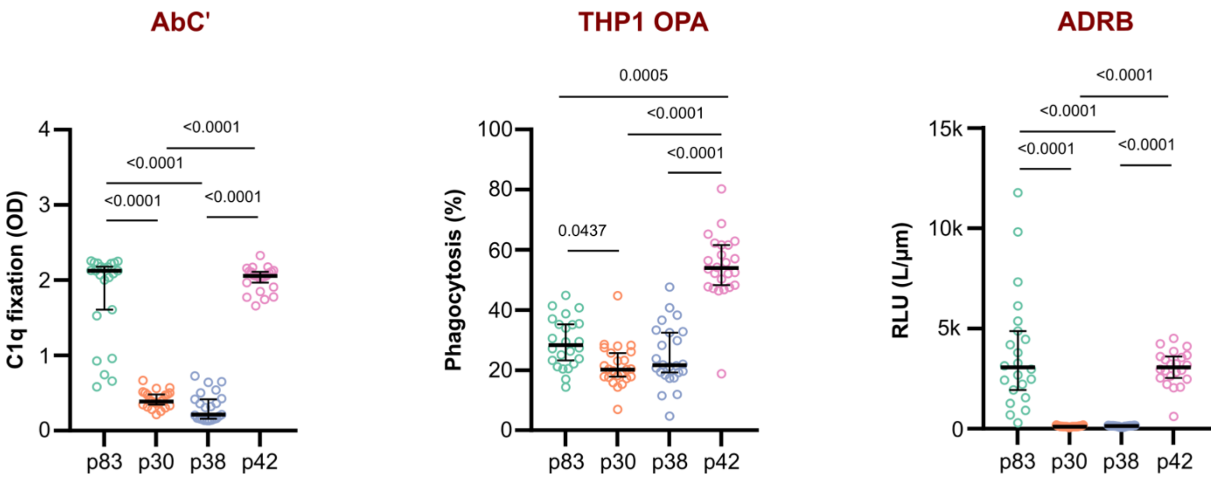

Supplementary Figure 4: The p83 and p42 subunit are primary targets of functional antibodies. Levels of post-immunization (D85) IgG-mediated AbC', THP1 OPA and ADRB were compared between MSP1 subunits. Error bars represent the median plus 95% confidence intervals. Statistical differences between timepoints were calculated using Friedmann test followed by Dunn's multiple comparisons test (Data file S3).

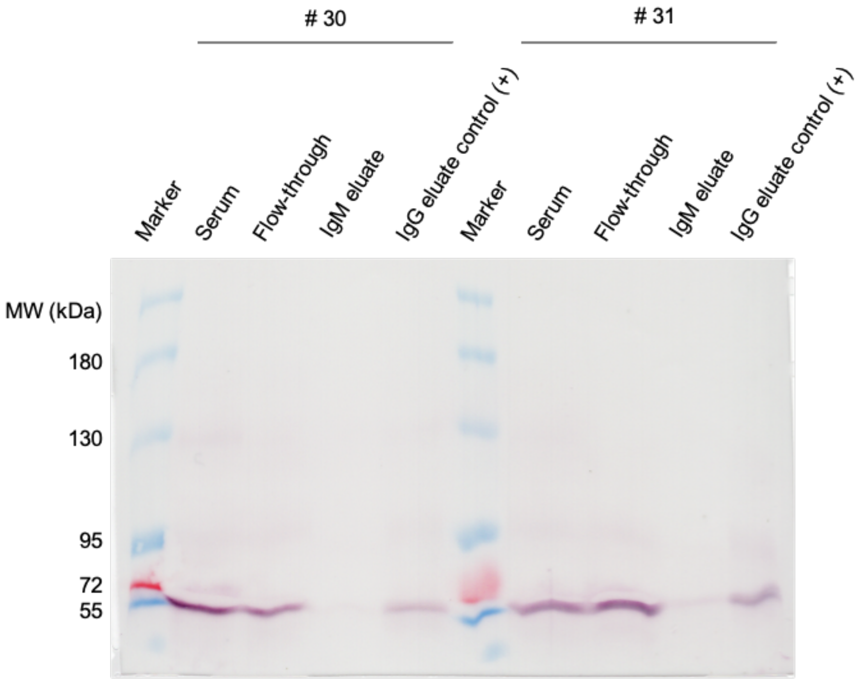

Supplementary Figure 5: Western Blot showing marginal IgG content in the IgM-eluted fraction. Reducing SDS-Page with loaded serum, flow-through and purified IgM and IgG samples Antibodies were detected using anti-human IgG-AP. Serum and flow-through of the IgM columns contain IgG, as expected. Eluate IgM served as negative control and does not contain IgG, as anticipated. Eluate IgG contains purified IgG.

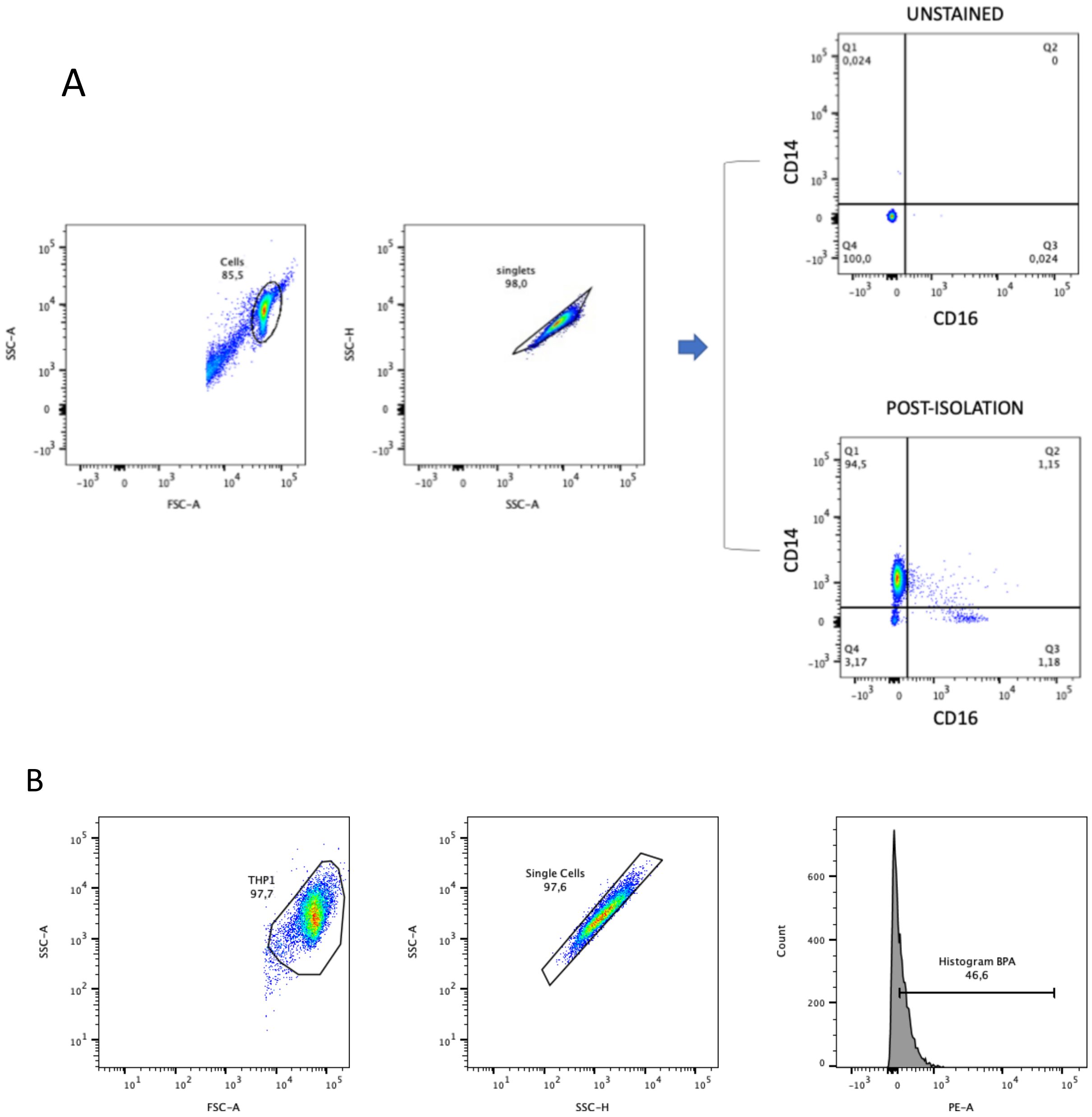

Supplementary Figure 6: Flow cytometry gating strategies: (A) Test for purity in the isolation of monocytes from peripheral blood: PBMCs were isolated from peripheral blood using density centrifugation and single cells were gated. A double staining $CD14^{+}/CD16^{+}$ was used to verify a monocyte ($CD14^{+}$) enrichment of at least 90%. **(B)** Gating strategy for OPA-THP1cells. Analysis of the data was performed using FlowJo software 10.2.

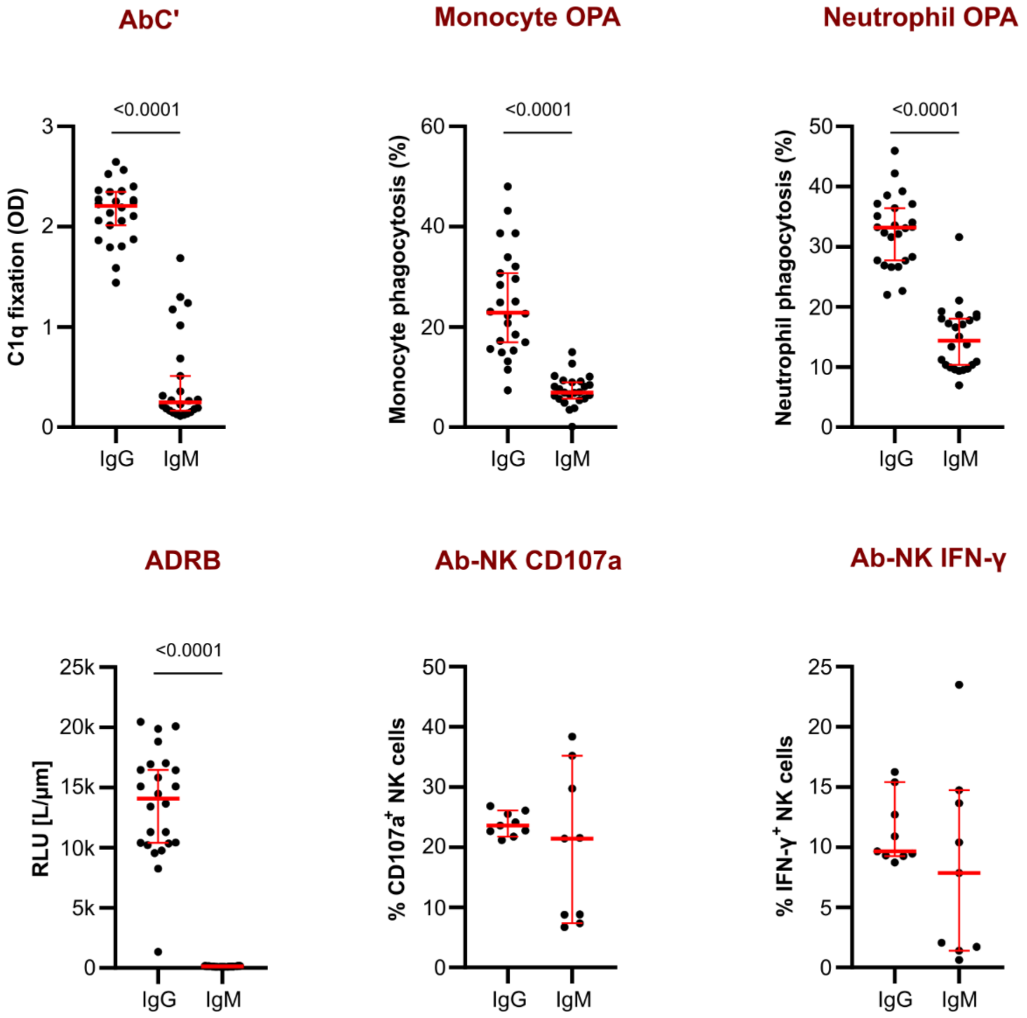

Supplementary Figure 7: Magnitude of Fc-mediated functional activity induced by IgG is higher compared to IgM. Levels of functional activity were compared between IgG and IgM post-immunization (D85). Error bars represent the median plus 95% confidence intervals. Statistical differences between IgG and IgM-mediated effector functions were calculated using Wilcoxon rank test.

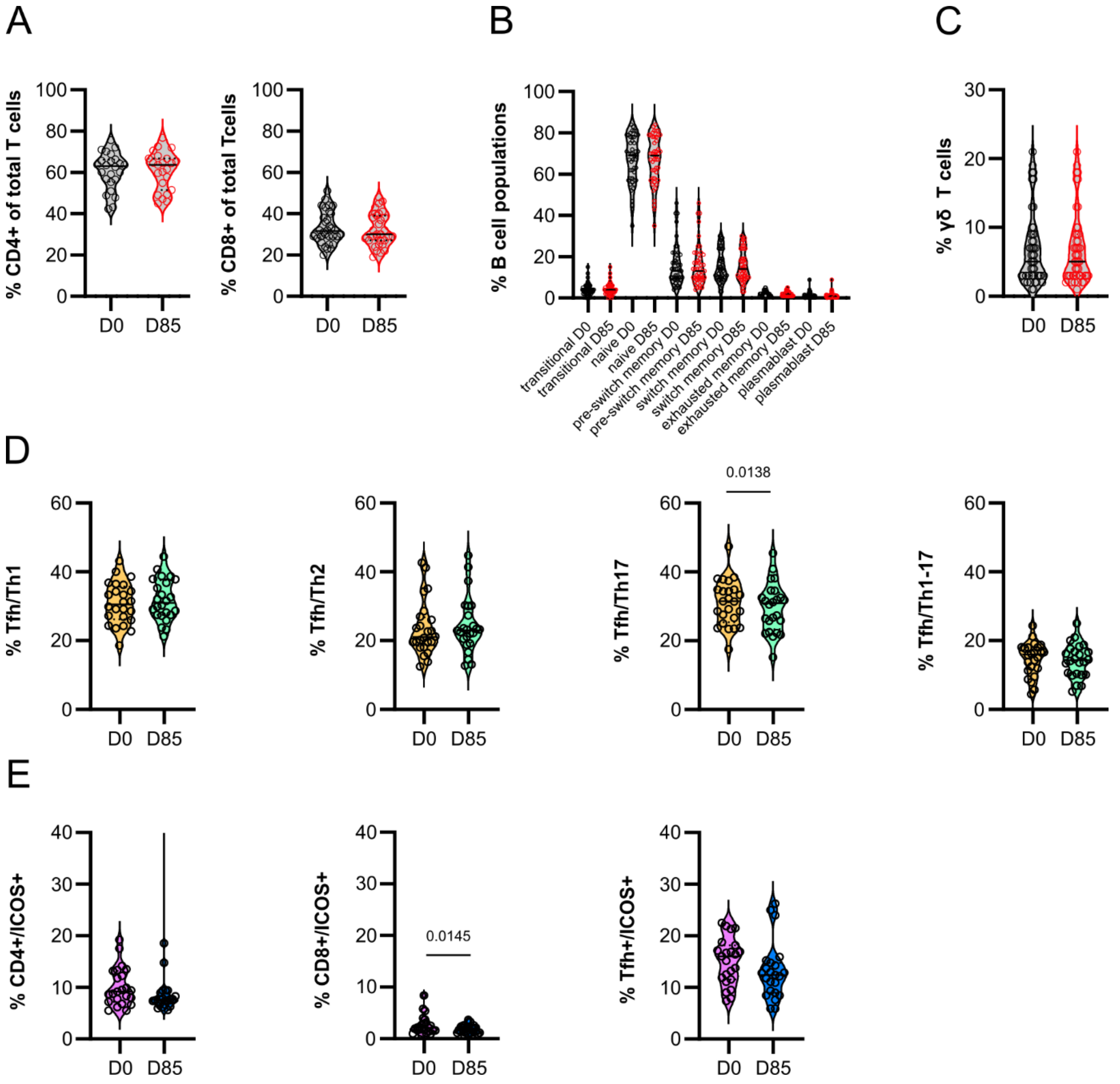

Supplementary Figure 8: Pair-wise comparison of a comprehensive flow cytometry panel of lymphocyte subpopulations before and after the trial. (**A-C**) No significant variation between day 0 and day 85 was observed for (**A**) $CD4^{+}$, $CD8^{+}$ T, for (**B**) the different B cell subsets explored and for (**C**) the γδ T cell population (**D**) For most Tfh subpopulations no significant changes were observed over time, only Tfh17 showed slight decrease in cell frequency. **(E)** No significant variation between day 0 and day 85 for the marker of activation ICOS except for the $CD8^{+}/ICOS^{+}$ subset (Data file S6).

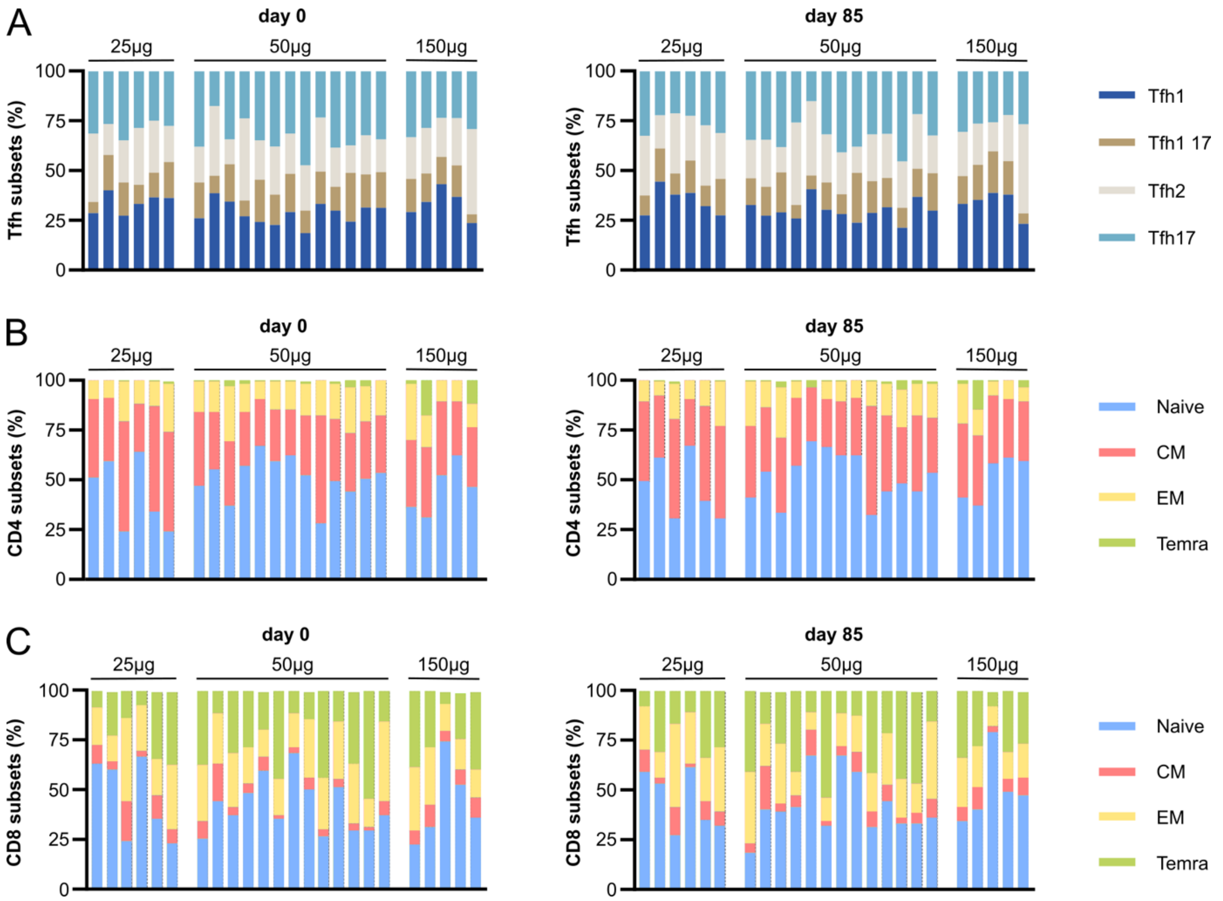

Supplementary Figure 9: Tfh and CD4^{+} and CD8^{+} memory subpopulations of T cells at the individual level and regarding the dose of immunization with *SumayaVac1*. (A) Tfh cell subsets (Tfh1, Tfh2 and Tfh1-17) at day 0 and day 85. **(B)** Different memory phenotypes (naïve, central memory (CM), effector memory (EM) and TEMRA) of CD4^{+} and CD8^{+} T cells at day 0 and day 85 (Data File S6).

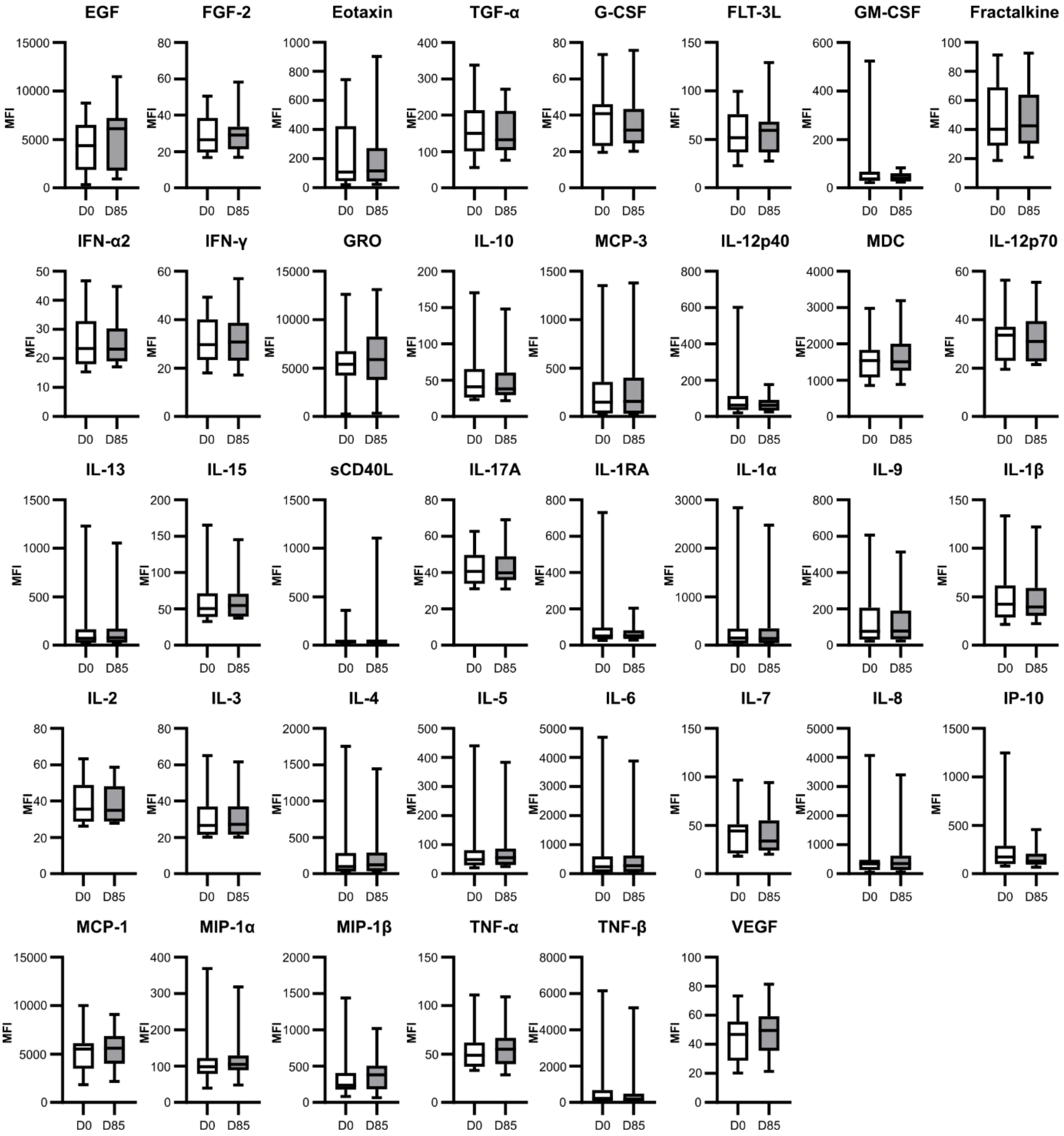

Supplementary Figure 10: Panel of 38 cytokines measured using bead-based multiplex assay. Cytokines EGF, FGF-2, Eotaxin, TGF-α, G-CSF, FLT-3L, GM-CSF, Fractalkine, IFN-α2, IFN-γ, GRO, IL-10, MCP-3, IL-12p40, MDC, IL-12p70, IL-13, IL-15, sCD40L, IL-17A, IL-1RA, IL-1α, IL-9, IL-1β, IL-2, IL-3, IL-4, IL-5, IL-6, IL-7, IL-8, IP-10, MCP-1, TNF-α, TNF-β and VEGF, comparing sera from day 0 (n=19) and day 85 (n=19) including all dosing groups. No differences were found in any of the cytokines tested (Data File S6).

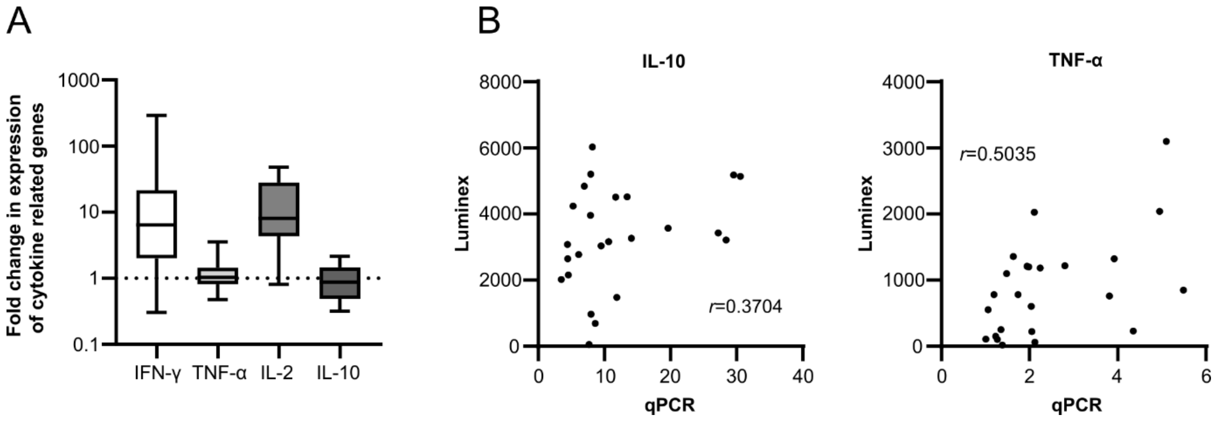

Supplementary Figure 11: RT-qPCR and correlation with Multiplex bead assay. (**A**) Fold change mRNA expression of cytokine related genes in PBMCs from vaccinees (day 85) compared to naïve PBMCs (day 0). The mRNA expression of IFN-γ, TNF-α, IL-2 and IL-10 for day 0 and day 85 were measured by real-time PCR and relative quantification of MSP-1-stimulated PBMCs to unstimulated PBMCs. Boxplots illustrate the medians, the 25th and 75th quartiles and the range (n=12). The positive fold change in IFN-γ and IL-2 mRNA gene expression shows the MSP1 specific memory immune response of vaccinated PBMCs. TNF-α and IL-10 mRNA gene expression does not change after vaccination. (**B**) Spearman correlations of relative gene expression measured by qPCR and Luminex of secreted TNF-α: *r*=0.50, *p*=0.0121, and IL-10, (non-significant) (Data file S6).

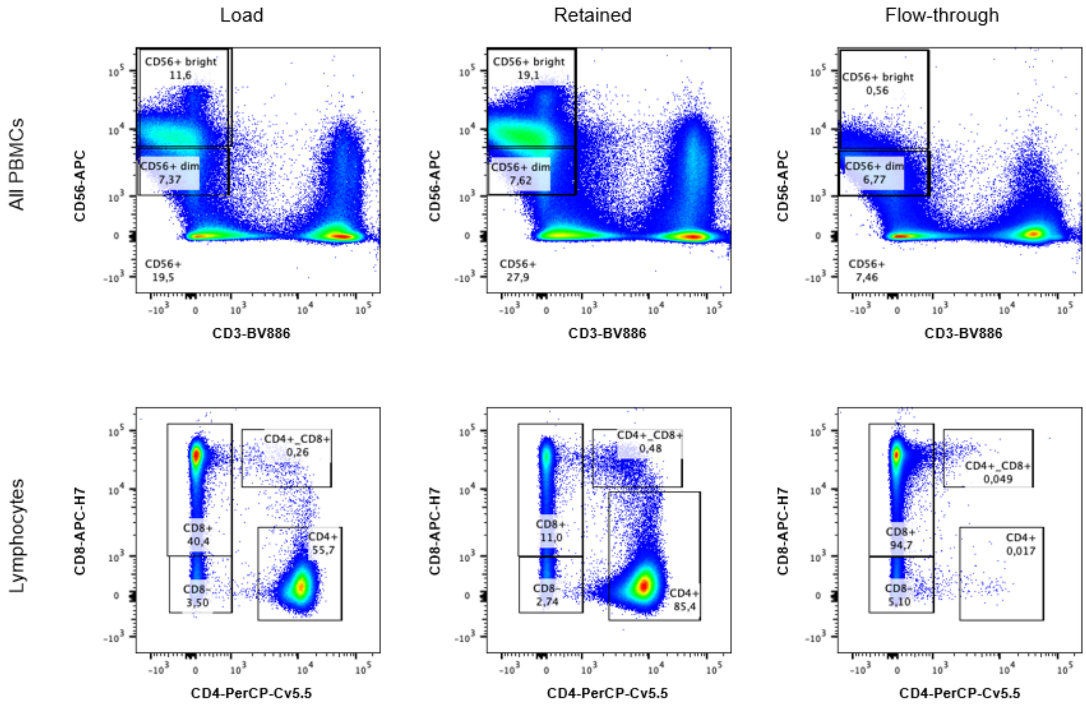

Supplementary Figure 12: CD8$^+$ T cells population enrichment for a CD8$^+$-specific ELISpot assay. Upper panel: different subpopulations of total PBMCs Percentage of CD56$^+$ cells pre and post magnetic sorting studied (load, retained and flow-through); please see materials and methods. Lower panel: different subpopulations of lymphocytes based on their expression of CD4+ and CD8+ T cells in the different fractions pre and post magnetic sorting (load, retained and flow-through) (Data File S7).

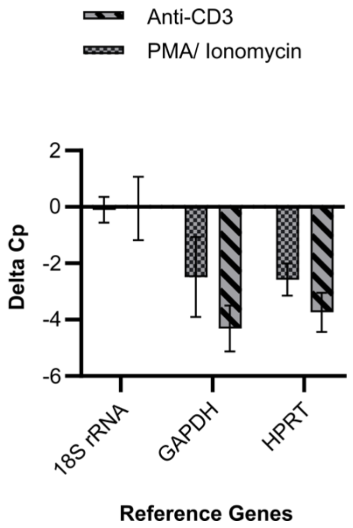

Supplementary Figure 13: Stability of the reference gene candidates 18S rRNA, HPRT and GAPDH. Evaluating the most stable reference gene for the relative quantification of cytokine related genes: PBMCs from 4 healthy volunteers were isolated, normalized against the unit cell amount and stimulated with PMA/Ionomycin and anti-CD3, respectively. The mRNA expression was detected by real-time PCR and data were analysed with the delta Cp method: Average ± SD from the delta Cp of the different reference genes. Delta Cp was calculated by subtracting the Cp from the unstimulated PBMCs from the one of the stimulated PBMCs.

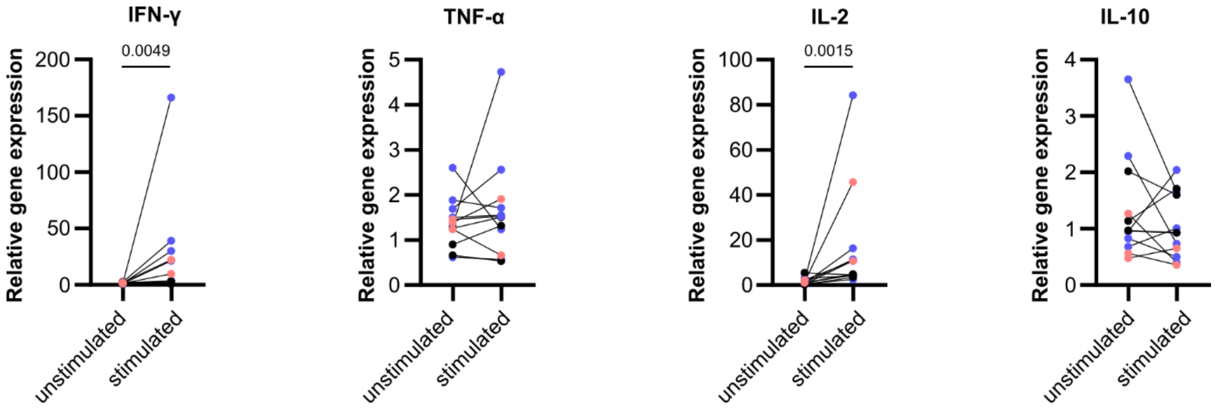

Supplementary Figure 14: Relative gene expression in cytokine related genes due to vaccination with SumayaVac-1 in in unstimulated and MSP-1 stimulated PBMCs. The mRNA expression of IFN-γ, TNF-α, IL-2 and IL-10 were measured by real-time RT-PCR. Relative quantification of day 85 PBMCs to the according day 0 PBMCs were done after 16h incubation in media (unstimulated) and MSP1-stimulation, respectively. One data point represents the gene expression in relative from day 85 to day 0, related to the corresponding culture condition. The dot colour represents vaccine dose (25µg; red dots, 50µg, blue dots and 150µg, black dots). A significant increase in IFN-γ and IL-2 mRNA expression in the MSP1-stimulated compared to the unstimulated PBMCs was observed. Statistical differences between timepoints were assessed using Wilcoxon rank test.

Median activity	Sera D85	IgG D85	IgM D85	IgG D182	IgM D182	IgG D210	IgM D210	IgG D365	IgM D365
ADRB-MSP1-D		14068.5 (1335-20461.5)	115 (84.5-184)	4953.5 (1823-9593)	108 (88.5-169)	16673 (11114-29332)	106 (79.5-136)	3393.5 (724-8291)	96.5 (81.5-120)
ADRB-3D7 merozoites**		884.6 (49.4-950.7)**							
ADRB-FCB1 merozoites**		798.0 (45.7-874.4)**							
AbC´-C1q MSP1-D	2.6862 (2.25-3.51)*	2.21 (1.44-2.64)	0.245 (0.11-1.68)	0.70 (0.21-2.24)	0.17 (0.07-0.4)	2.01 (1.11-2.45)	0.25 (0.17-0.92)	0.43 (0.08-1.59)	0.12 (0.08-0.39)
AbC´-C1q MSP1-F	1.3 (0.35-2.75)*								
AbC´-C3b		2.2625 (1.59-2.49)	2.1735 (0.15-2.60)						
AbC´-C5-C9		3.4995 (2.99-3.86)	2.727 (0.79-3.18)						
OPA THP1/Mono MSP1-D	29.35 (2.91-39.4)*	22.85 (7.32-48)	6.9 (0.11-14.95)	6.21 (2-21)	5.743 (4.01-9.07)	13.28 (7.12-36.6)	7,23 (4.78-14.45)	7.06 (2.47-26.1)	6.144 (4.75-12.9)
OPA THP1/Mono MSP1-F	30.125 (10.75-41.2)*								
OPA Neutro		33.17 (22-45.95)		21.15 (8.95-28.3)		34.55 (23.6-41)		18.35 (9.79-31.3)	
Ab-NK CD107a		24.1 (21.15-27.1)	21.45 (6.71-38.35)	23 (18.4-25.2)	6.99 (5.76-23)	24,25 (21.3-27.7)	14.98 (7.37-36.1)	22.55 (18.9-24.8)	6.67 (4.94-13.95)
Ab-NK IFN-γ		10.91 (8.74-15.4)	7.85 (0.61-23.5)	10.9 (6.27-15.8)	1.03 (0.47-9.26)	12.6 (8.48-18.8)	4.46 (1.58-14.55)	10,3 (5.2-13)	1.28 (0.53-4.17)

Supplementary Table 1: Side-by-side comparison of the median activity of Fc-mediated effector functions of IgG and IgM and the Fold change at day 85 between IgG (1mg/mL) when compared to IgM (2 mg/mL). (*) Data using monocytes. (**) ADRB results from Blank et al., npj vaccines 2020, using isoluminol.
